# Simulation-Based Learning Supported by Technology to Enhance Critical Thinking in Nursing Students: Scoping Review

**DOI:** 10.2196/58744

**Published:** 2025-02-18

**Authors:** Hege Vistven Stenseth, Simen A Steindal, Marianne Trygg Solberg, Mia Alexandra Ølnes, Anne Lene Sørensen, Camilla Strandell-Laine, Camilla Olaussen, Caroline Farsjø Aure, Ingunn Pedersen, Jaroslav Zlamal, Jussara Gue Martini, Paula Bresolin, Silje Christin Wang Linnerud, Andréa Aparecida Gonçalves Nes

**Affiliations:** 1 Department of Graduate Studies Faculty of Nursing Lovisenberg Diaconal University College Oslo Norway; 2 Institute of Nursing Faculty of Health Sciences VID Specialized University Oslo Norway; 3 Department of Health and Nursing Science Faculty of Health and Sports Sciences University of Agder Grimstad Norway; 4 Novia University of Applied Sciences Turku Finland; 5 Nord University Bodø Norway; 6 Clinical Simulation Center Vestfold Hospital Trust Tønsberg Norway; 7 Postgraduate Program in Nursing Universidade Federal de Santa Catarina Florianópolis Brazil; 8 Department of Caring and Ethics Faculty of Health Sciences University of Stavanger Stavanger Norway

**Keywords:** critical thinking, simulation-based learning, technologically supported simulation-based learning, nursing education, nursing students, review

## Abstract

**Background:**

Critical thinking is a crucial skill in the nursing profession and must be fostered through nursing education. Simulation-based learning (SBL) with technological modalities is a pedagogical approach to enhance critical thinking skills for nursing students. The use of technology in SBL to achieve critical thinking skills is diverse. No previous scoping review has systematically mapped studies on SBL supported by technology to enhance critical thinking in nursing students.

**Objective:**

This scoping review aimed to systematically map research on the use of SBL supported by technology to enhance critical thinking in nursing students.

**Methods:**

This scoping review was conducted according to the framework by Arksey and O’Malley and was reported according to the PRISMA-ScR (Preferred Reporting Items for Systematic Reviews and Meta-Analyses extension for Scoping Reviews) guidelines. A systematic, comprehensive literature search was performed in the LILACS, ERIC, MEDLINE, Embase, PsycINFO, and Web of Science databases in 2021 and repeated in 2023 and 2024. Pairs of authors independently assessed titles, abstracts, and full-text papers and extracted data from the included studies. The data underwent summative and thematic analysis and were categorized according to the findings.

**Results:**

In total, 4 main categories of technology applied in SBL were identified: computer-based simulations, human-patient simulators, virtual reality or immersive virtual reality, and others. The findings revealed a shift across time in the technology used for SBL to enhance critical thinking, from human patient simulators to computer-based simulations. A dominant part of the included studies published after 2018 (21/44, 48%) incorporated a combination of asynchronous and synchronous learning activities. The theoretical foundation of the studies revealed a range of scientific theories and conceptual frameworks and models. Enablers of or barriers to the enhancement of critical thinking skills in nursing students were identified within the following themes: affinity for and availability of technology, realism, accessibility, engagement and motivation, validation, return on investment, and enhanced critical thinking through SBL using technology.

**Conclusions:**

There has been a noticeable shift in the technology and use of technology in SBL. Descriptions of the applied technology and pedagogical considerations are pivotal for comparing or synthesizing research results. There has been a trend toward a blended educational approach combining synchronous and asynchronous learning activities. User technological proficiency and the perceived quality of the technology are imperative in the development of critical thinking. Realism, engagement, and motivation play pivotal roles in the enhancement of critical thinking in technologically supported SBL. The establishment of robust theoretical foundations of research and standardized research practices will strengthen the evidence obtained from the research conducted.

## Introduction

### Background

Critical thinking skills are vital for professional nurses to perform nursing care in a safe, evidence-based manner. Nurses need to be able to analyze, summarize, and evaluate information and initiate appropriate actions. Critical thinking skills empower nurses to manage the uncertainties inherent in nursing practice, thus contributing to safe, effective care across diverse clinical settings [1-4]. Critical thinking includes purposeful and self-regulatory judgment in a process of interpretation, analysis, evaluation, and inference [5]. Critical thinking also includes evidential, conceptual, methodological, criteriological, or contextual considerations based on the relevant judgment [5]. Critical thinking is driven by internal motivation and characterized by reflection, self-monitoring, and self-correction. This process cultivates a reflective judgment on what to do, believe, or make sense of in any given context [6]. However, critical thinking is a concept that has no established or agreed upon definition [7]. As Cassum et al [8] have pointed out, individuals may often define critical thinking based on their own understanding and choose to emphasize certain aspects of critical thinking. Several studies have pointed out that critical thinking is used synonymously with other concepts, such as clinical decision-making, analytical thinking, clinical judgment, critical judgment, creative thinking, problem-solving, reflective thinking, and diagnostic reasoning or higher-order thinking [9,10]. This is mirrored in a study by Geng [11], who identified 64 definitions of critical thinking. The common characteristics of critical thinking in these definitions were judgment, argument, questioning, information processing, problem-solving, and meta-cognition. These characteristics can be found both in definitions of critical thinking, for example, in the definition by Facione [5], and in definitions of other concepts, such as clinical reasoning, decision-making, or clinical judgment [12,13]. Although achieving consensus on the definition of critical thinking poses challenges for identifying studies related to this concept, there is no doubt about its vital importance in the field of nursing.

To facilitate the development of critical thinking skills in nursing students, educators could encourage active student participation in the learning process. Simulation-based learning (SBL) is one pedagogical approach based on principles of active learning [1-3]. Bland et al [14] have conceptualized and defined SBL in nursing education as “a dynamic process involving the creation of a hypothetical opportunity that incorporates an authentic representation of reality, facilitates active student engagement and integrates the complexities of practical and theoretical learning with opportunity for repetition, feedback, evaluation and reflection.” SBL offers possibilities for experiential learning and deliberate practice through the integration of theoretical and practical knowledge, application of skills, and development of the ability to reflect and give and receive feedback [15]. Furthermore, SBL aims to replicate features of clinical practice that require learners to use critical thinking and clinical reasoning, involving cognitive, psychomotor, and affective skills [16,17].

Technological solutions for SBL in nursing education are rapidly evolving, and different simulation modalities are applied according to pedagogical and practical considerations [15,18]. SBL in nursing education can be divided into 2 main categories: SBL in the physical learning environment and SBL in the virtual learning environment. The physical learning environment is typically represented by simulation laboratories within universities but can include SBL in the clinical environment and context as well. The virtual learning environment refers to a digital or computerized environment that provides opportunities for independence in learning and diverse approaches to interacting with technology and others within the virtual world [19]. The types of simulation technology or equipment used as part of the simulation activity are often referred to using the term *simulation modality* [20]. The modality is the platform and resources used to support the simulated experience and may incorporate a variety of technologies [21,22]. One common modality for SBL is human-patient simulators (HPSs), which are advanced physical simulators with humanlike features and responses driven by computers [22]. Other modalities often applied in virtual learning environments can be categorized as technology-enhanced simulation (TES). TES is a blanket term for SBL with direct or assisted interaction with an electronic medium presented through computers or other technology that provides learners with a virtual environment in which they complete certain tasks, use information, assess, make clinical decisions, and observe results [20]. TES modalities include computer-assisted simulation, serious games, and computer simulation games, collectively referred to as computer-based simulation (CBS). CBSs are interactive simulation games in which learners are assigned roles, typically as the nurse, and nursing students are involved in a scenario targeted to interpreting information and problem-solving [23]. Virtual reality (VR) and immersive VR (IVR) are the most recently introduced TES modalities, with different levels of immersion. These modalities seek to replicate specific clinical settings in a virtual environment. IVR distinguishes itself from other VR technologies by using special headsets that immerse the participants in a virtual world [24-26]. IVR experiences are often designed to promote specific learning outcomes in scenarios with patient-nurse interactions in a suitable clinical environment [27].

Several previous reviews have examined the prevalence and effectiveness of SBL to enhance critical thinking in nursing education [16,18,28-30]. SBL is the predominant learning activity that uses digital tools in nursing education [18]. A scoping review on teaching strategies for the development of clinical reasoning in advanced nursing clinical practice reported that SBL is the most frequently encountered teaching strategy [28]. Systematic reviews have examined the effectiveness of SBL on clinical reasoning and critical thinking skills in nursing education [16,29,30]. These reviews have determined that there is insufficient evidence to form definitive conclusions on the effect of SBL on clinical reasoning and critical thinking. However, the use of technology to enhance critical thinking in SBL has not been thoroughly addressed in these reviews.

Reviews have also examined the prevalence of SBL supported by technology and compared SBL that uses different technologies or SBL that uses technology versus traditional teaching methods [15,26,31,32]. A systematic review and meta-analysis of 12 randomized controlled trials concluded that VR offers higher potential than conventional teaching methods in nursing education for advancing outcomes of overall satisfaction, theoretical knowledge, and practice proficiencies without providing an advantage in enhancing critical thinking skills [31]. This review limited the examination to randomized controlled trials, excluding studies with other research designs [31]. Another systematic review examined game-based learning in undergraduate nursing education and found serious games to be the most used game type. Game-based learning was effective in achieving learning outcomes, particularly in the cognitive domain [32]. However, critical thinking was not a specific outcome, and only mixed methods studies were included in the review. In addition, a scoping review explored the use of virtual simulation (VS), an interactive recreation of real-life clinical scenarios using computer technology, to enhance diagnostic reasoning in health care professional education. VS was found to be at least as effective if not superior to traditional SBL, but only 3 of the 12 included studies were related to nursing education [26]. Another literature review also supported the effectiveness of VS in improving skills, learning, and critical thinking in nursing education [15]. However, critical thinking was the least explored outcome, the literature search was limited to 2 databases, and solely English-language papers were included.

Moreover, reviews have investigated the effectiveness of SBL in enhancing critical thinking in nursing education incorporating technology [33,34]. A systematic review and meta-analysis focused on VS as a tool to enhance clinical reasoning in nursing education and found that VS can improve clinical reasoning skills [34].

A systematic review comparing VR to traditional SBL practices concluded that the effectiveness of VR on clinical reasoning educational outcomes was similar or superior to that of traditional methods, although the evidence is limited [33]. A meta-analysis on technology in the SBL learning environment for the development of complex skills such as critical thinking across different educational domains stated that recent technologies can greatly enhance the effect of SBL [4]. A qualitative literature review explored nursing students’ experiences with virtual screen-based simulations and found that a comfortable atmosphere; engagement and feedback; and improved skills, including critical thinking and decision-making skills, positively impacted learning in VS. Negative experiences with VS stemmed from the need for more support, poor design, and lack of authenticity [35].

### Objectives

In nursing education, SBL supported by technology is increasingly applied as a learning strategy given the collective demand for active and flexible educational approaches and the growing interest and need for technological solutions [36]. Understanding the enablers and barriers associated with these solutions may be of great value for selecting suitable technological modalities for SBL to promote the development of critical thinking. Previous reviews have not comprehensively examined the range and use of technology for supporting SBL in enhancing nursing students’ critical thinking skills. Therefore, there is a need for a broad, comprehensive literature review, such as a scoping review, including scientific papers in several languages and that use diverse research methods. Such a review is important to summarize the scope and variety of published peer-reviewed studies in this area while also identifying potential gaps in the existing peer-reviewed studies [37]. To the best of our knowledge, no scoping review has investigated the variety and use of technology in SBL for enhancing nursing students’ critical thinking skills. Consequently, this scoping review aimed to systematically map studies on the use of SBL supported by technology to enhance critical thinking in nursing students.

## Methods

### Overview

This scoping review was conducted according to the methodological framework by Arksey and O’Malley [37], which includes the following steps: (1) identifying the research questions (RQs); (2) identifying relevant studies; (3) selecting studies; (4) charting the data; and (5) collating, summarizing, and reporting the results. The PRISMA-ScR (Preferred Reporting Items for Systematic Reviews and Meta-Analyses extension for Scoping Reviews) guidelines were followed for the reporting of this review [38,39]. The research protocol was published in *JMIR Research Protocols* [40], and the deviations from the protocol are described in Multimedia Appendix 1.

### Identifying the RQs

The following RQs were asked:

1. What variety of technology is used in SBL to enhance critical thinking skills in nursing education? (RQ 1)
2. How is technology used in SBL to enhance critical thinking skills in nursing education? (RQ 2)
3. What barriers and enablers do nursing students report in the use of technology in SBL to enhance critical thinking? (RQ 3)

### Identifying Relevant Studies

The Sample, Phenomenon of Interest, Design, Evaluation, and Research Type framework determined the inclusion and exclusion criteria [41] (Textbox 1).

**Eligibility criteria for inclusion of the selected studies.**

**Inclusion criteria**

- Sample: papers focused on undergraduate and postgraduate nursing students
- Phenomenon of interest (the main aim of this scoping review was to systematically map studies on the use of simulation-based learning [SBL] supported by technology to enhance critical thinking in nursing students. As such, it is warranted to use critical thinking as a concept interchangeably with other concepts): SBL supported by technology to stimulate critical thinking, clinical decision-making, analytical thinking, creative thinking, problem-solving, reflective thinking, diagnostic reasoning, clinical reasoning, or clinical judgment in educational or institutional contexts; SBL supported by technology, including human patient simulator–based modalities, virtual reality (VR), immersive VR, virtual simulation, augmented reality, or computer-based simulation
- Design: studies with quantitative, qualitative, or mixed methods designs
- Evaluation: undergraduate and postgraduate nursing students’ perspectives and experiences regarding the use of technology in SBL to enhance critical thinking or similar concepts
- Research type: studies of any research type published in Portuguese, Spanish, English, Norwegian, Swedish, or Danish in peer-reviewed journals

**Exclusion criteria**

- Sample: papers focused health care students other than nursing students
- Phenomenon of interest: SBL that does not use technology; SBL that uses technology but is not related to critical thinking or similar concepts; SBL in clinical practice not related to education
- Evaluation: nurse educators’ perspectives and experiences regarding the use of technology in SBL to stimulate critical thinking
- Research type: case studies, case reports, clinical guidelines, all types of reviews, master’s and PhD theses, conference proceedings and abstracts, letters, comments, discussions, editorials, and book chapters

### Selecting Studies

A systematic search was conducted in CINAHL, ERIC, Embase, LILACS, Ovid MEDLINE, PsycINFO, and Web of Science on June 28, 2021. The database search was updated on March 17, 2023, and November 13, 2024. Each database was searched from its inception.

The search strategy in Ovid MEDLINE, using Medical Subject Headings and text words, was designed by an experienced research librarian (MAØ) in collaboration with the rest of the research team and encompassed 3 elements: SBL, technology, and nursing students and nursing education. A second research librarian (Kari Larsen Mariussen) reviewed the search strategy using the Peer Review of Electronic Search Strategies Checklist [42]. The search strategy in Ovid MEDLINE is outlined in Multimedia Appendix 2. In addition, we manually examined the reference lists of the included papers to determine whether any studies mentioned in those references were relevant to our review. Moreover, we conducted forward citation searching using the Google Scholar platform to identify relevant studies that had cited the included papers.

The identified citations were exported to EndNote (Clarivate Analytics) for removal of duplicates using the method described by Bramer et al [43]. The citations were randomly divided into 8 groups and transferred to the web application Rayyan (Qatar Computing Research Institute) [44] for storage, organization, and blinding of the study selection process. HVS and AAGN screened the titles and abstracts of 10% of the papers to pilot-test the eligibility criteria and deemed that the eligibility criteria did not require modification. In total, 8 pairs of authors and nonauthor contributors (HVS and CFA, SCWL and SAS, MTS and JZ, Andrea Mohallem and Fernando Riegel [nonauthors], PB and JGM, ALS and CS-L, CO and HVS, and IP and AAGN) performed the study selection process. Pairs of authors independently assessed whether titles and abstracts and then full-text articles met the eligibility criteria. When there was any doubt regarding inclusion, a third author (HVS or AAGN) independently assessed the full-text paper, and the decision was based on a negotiated consensus. The second updated search in 2024 was conducted by 2 pairs of authors (HVS and SAS and AAGN and MTS) consistent with the previous procedure for study selection.

### Charting the Data

A standardized data collection form was developed in Microsoft Excel for data extraction from the included papers, including the following data: authors, year, and country; aim; sample; design; technology; simulation procedures; scenario design; and results related to the RQs. HVS and AAGN piloted the data collection form on 5 of the included papers, and some small revisions were made. Pairs of authors (Andrea Mohallem and Fernando Riegel, SCWL and SAS, PB and JGM, CO and CFA, IP and AAGN, JZ and MTS, and ALS and CS-L) extracted data from the full-text papers. One author extracted the data, and the other checked their accuracy against the papers. Disagreement or uncertainties among pairs of authors were resolved through an independent assessment by HVS, and the agreement was based on negotiated consensus. The extracted data were also checked by the first (HVS) and last (AAGN) authors to ensure quality and consistency in data extraction.

### Collating, Summarizing, and Reporting the Results

Data from the included papers regarding RQ 1 and RQ 2 were analyzed by HVS and AAGN using summative analysis. Regarding RQ 3, data were analyzed using an inductive approach to organize the results thematically, a method previously used in scoping reviews [45-47]. HVS and AAGN read the extracted data several times to identify patterns of similarities and differences related to the RQ, and the patterns were organized into subthemes and themes using a low level of interpretation and abstraction. An example of inductive thematic analysis for RQ 3 includes participants highlighting the ability to attend the SBL regardless of time and place, which was categorized under subtheme Flexibility and Theme Affinity for technology. Uncertainties of owns performance was categorized under subtheme Need for feedback and theme Validation.

SAS provided feedback on the preliminary thematic groupings. The data were then analyzed deductively from the perspective of the 6 dimensions (system quality, information quality, service quality, use and intention to use, user satisfaction, and net benefits) of the DeLone and McLean (D&M) [48,49] model of information system (IS) success. These themes were sorted accordingly and subsequently positioned within the predetermined categories of the framework of the D&M IS Success Model through deductive analysis. This model aims to evaluate the success of an IS and suggests that its dimensions are interrelated and influence each other [48]. The model has been widely used in research and practice to assess the success of various types of ISs, including enterprise systems, e-commerce platforms, and educational technology [19,49,50]. Studies exploring learners’ (nursing students’) experiences with technology have provided valuable insights into how technology affects their ability to develop critical thinking skills. User satisfaction, a key indicator of IS success, served as the basis for evaluating the technology’s effectiveness [50]. The reported enablers and barriers for critical thinking enhancement through SBL using technology were sorted according to the results of the inductive analysis. The outcomes of all the studies were related to the development of critical thinking among nursing students through learning activities with technologically supported SBL. Therefore, the outcomes would be synonymous with learning and the achievement of learning outcomes.

## Results

### Overview

**Figure 1:**
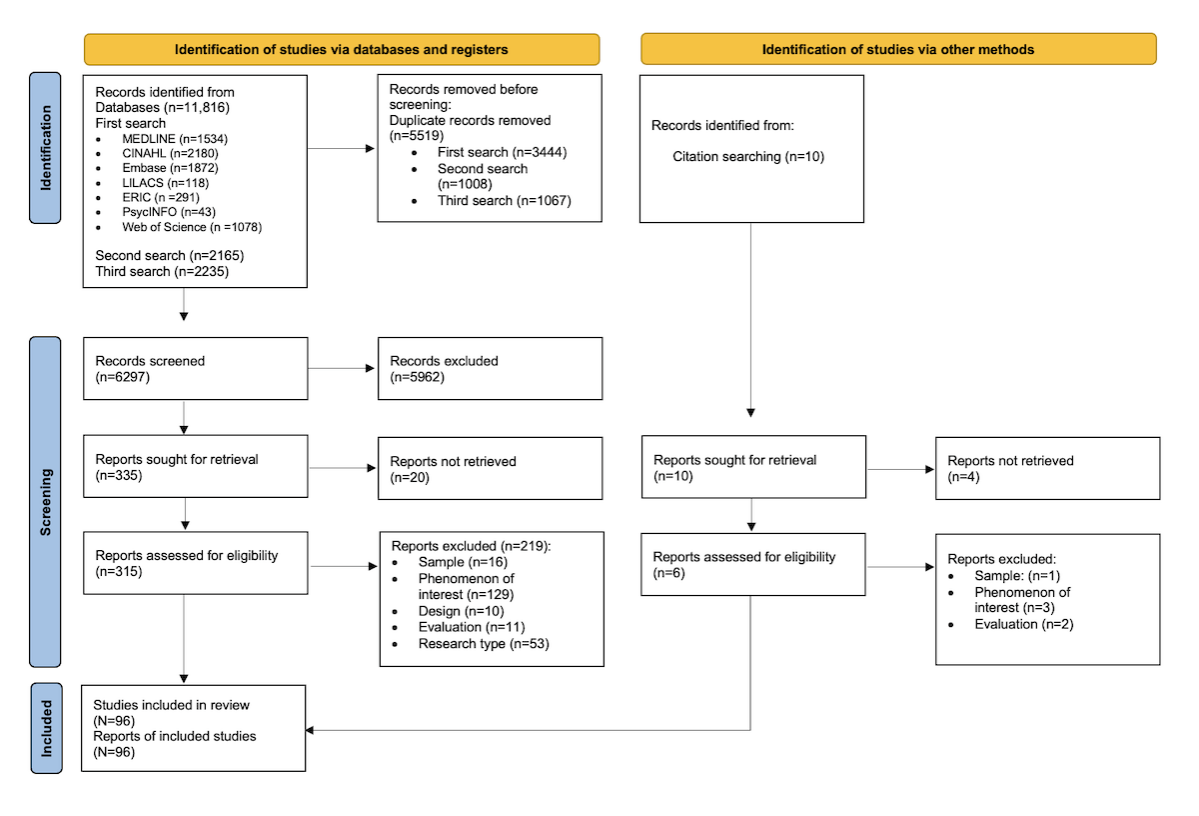
PRISMA (Preferred Reporting Items for Systematic Reviews and Meta-Analyses) 2020 flow diagram of the study selection process for this scoping review.

After the screening of 6297 records and 315 (5%) full-text papers, a total of 96 (1.52%) studies were included. Citations with additional information are listed in Multimedia Appendix 3 [51-146]. The study selection process and reasons for exclusion of full-text papers are presented in Figure 1 [39].

### Sociodemographic Findings

The studies originated from 20 countries: the United States (42/96, 44%) [51-92], South Korea (12/96, 12%) [93-104], Australia (6/96, 6%) [105-110], China (6/96, 6%) [111-116], Turkey (6/96, 6%) [117-122], Canada (4/96, 4%) [123-126], Finland (3/96, 3%) [127-129], Saudi Arabia (3/96, 3%) [130-132], Taiwan (3/96, 3%) [133-135], France (1/96, 1%) [136], Hong Kong (1/96, 1%) [137], Indonesia (1/96, 1%) [138], Iran (1/96, 1%) [139], Israel (1/96, 1%) [140], Lebanon and Jordan (1/96, 1%) [141], Norway (1/96, 1%) [142], Portugal (1/96, 1%) [143], Sweden (1/96, 1%) [144], Thailand (1/96, 1%) [145], and the United Kingdom (1/96, 1%) [146].

The years of publication ranged from 1986 to 2024. Most studies (66/96, 69%) used a quantitative design [51, 52, 54-69, 71-74, 78, 86, 89-96, 98-104, 111, 114-119, 121, 127-130, 132-137, 139-141, 143-146]. A mixed methods, multimethod, or blended research approach was applied in 20% (19/96) of the studies [53,77,83-85,87,97,106,107,109,110,112,113,122-126,138], and 11% (11/96) of the studies incorporated a qualitative design [58,75,79,81,82,88,105,108,120,131,142].

Most studies (92/96, 96%) included undergraduate nursing students. The studies also included Master of Science nursing students [83]; nurse practitioner master’s degree students [86]; postgraduate nurse anesthesia students [82]; and nursing students preparing at the diploma, associate, and baccalaureate levels [54]. The sample sizes ranged from 8 to 675 participants. The reported ages of the participants (73/96, 76% of the studies) ranged from 15 to 56 years. All studies that reported demographic data on gender (67/96, 70%) had a majority of female participants, ranging from 57% to 100% except for one study that reported 52% male participants [131]. A total of 23% (22/96) of the studies did not report demographic data regarding age or gender [59, 63, 66, 67, 73, 78, 80, 82, 83, 85, 88-91, 97, 98, 101, 105-107, 129, 146]. In total, 10% (10/96) of the studies reported age but not gender [76, 96, 102, 103, 109, 126-128, 133, 134], and 4% (4/96) of the studies reported data on gender but not on age [77,112,121,137].

### Theoretical Foundations and Measurement of Critical Thinking

More than half (56/96, 58%) of the studies [51-55, 61, 63, 65, 69, 74-77, 80, 81, 83-92, 97, 100, 102-105, 107, 109, 111, 112, 114, 115, 118-126, 128-131, 135-137, 139, 140, 145] presented a theory or conceptual framework as a foundation for the study conducted. Scientific theories such as learning theory (24/96, 25%), nursing theory (7/96, 7%), and philosophical-methodological theory (2/96, 2%) were applied alone or in combination 33 times across the studies. Specific theoretical models or conceptual frameworks were presented as the theoretical foundation alone or in combination 36 times across the studies.

Most studies (54/96, 56%) used one or more validated instruments to evaluate the development of critical thinking skills or equivalent concepts [51, 55-57, 60, 64, 65, 67, 69, 72, 74, 76, 77, 84-87, 91-93, 95-104, 111-119, 121-124, 126, 129, 132-137, 139, 141, 144]. Across these 54 studies, 42 validated instruments were used to assess outcomes. The California Critical Thinking Disposition Inventory, including its Chinese version, was used in 13% (7/54) of these studies to measure critical thinking. The Lasater Clinical Judgment Rubric was the second most frequently used instrument, applied in 11% (6/54) of the studies. The Critical Thinking Disposition Tool was used in 9% (5/54) of the studies, whereas the Simulation Effectiveness Tool–Modified was used in 7% (4/54) of the studies, including one instance of using the Turkish version. Notably, 29 instruments were used in only one study each. A detailed overview is provided in Multimedia Appendix 3.

### Range and Features of the Technology Applied in SBL for Enhancing Nursing Students’ Critical Thinking (RQ 1)

The technology in SBL to enhance critical thinking was divided into 4 main categories. The first category of applied technology was CBS (44/96, 46%) [52, 53, 55, 59, 67, 76, 78, 83, 85, 86, 89, 90, 93, 96, 97, 99, 101, 104, 105, 108, 109, 111, 114, 117-125, 127, 128, 130-133, 136-140, 143]. A wide variety of terms was used in the studies, and details are presented in Multimedia Appendix 3. The second category of applied technology was HPSs (26/96, 27%) [51, 57, 60, 62-66, 68-73, 80-82, 84, 87, 91, 94, 100, 107, 141, 145, 146]. The third category of applied technology in SBL was VR or IVR (7/96, 7%) [75,77,95,98,103,110,126].

The fourth category comprised studies (19/96, 20%) that used several or combinations of technologies or technology not consistent with the first 3 categories. In these studies, technology was applied through a combination of CBS, an HPS and case studies [116], a combination of CBS and IVR simulation [115,129], a combination of CBS and simulated electronic health records [61], multimedia technologies combined with an HPS [58], videotaped vignettes combined with an HPS [54], FaceTime technology [79], an interactive videodisc system [134], telehealth simulation and real-time video and audio technology [142], telehealth simulation and an HPS [135], mixed reality [106], a combination of CBS and HPSs [56,102,110,112,113,144], a combination of an HPS and interactive case studies [74], and a VS designed in Microsoft PowerPoint [88]. An overview is provided in Table 1.

**Figure 2:**
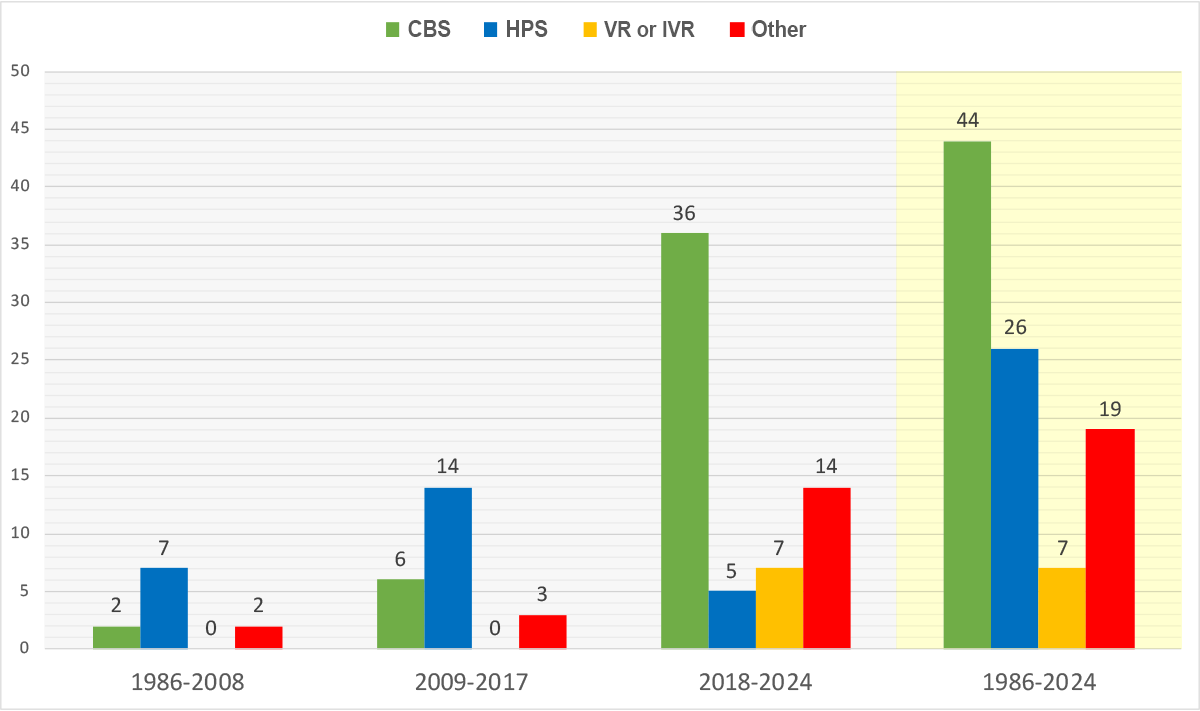
Evolution of the applied technology in simulation-based learning to enhance critical thinking from 1986 to 2024. CBS: computer-based simulation; HPS: human-patient simulator; IVR: immersive virtual reality; VR: virtual reality.

The analysis revealed an evolution of the technology used in the included studies across time, from most studies (21/32, 66%) based on HPSs as the modality before 2018 to TESs using CBS as the major modality category in studies after 2018 (Figure 2).

The technology applied was diversely described in the studies. Some studies using HPS technology (25/96, 26%) gave descriptions of features and functions, but these studies generally provided limited description of the HPSs, which were often described only by the HPS’s name and producer [51, 62-64, 68-71, 81, 82, 87, 91, 94, 96, 100, 102, 107, 113, 116, 135, 141, 144-146]. For CBS and VR or IVR, there was generally a richer description of the technology used, with descriptions of features that could contribute to enhancing critical thinking in SBL through learning. Several studies (23/96, 24%) used commercially developed technology featuring preprogrammed scenarios with virtual patients and VS [52, 53, 59, 61, 67, 75, 83, 86, 89, 92, 93, 96, 99, 105, 106, 109-117, 121, 122, 125, 126, 131, 133, 143, 144]. Some studies (16/96, 17%) investigated technological solutions or software for CBS, VR, or IVR that was self-developed or developed in a cooperation between expert nursing educators and IT engineers [78, 85, 88, 90, 98, 101-104, 108, 118, 120, 124, 130, 133, 136]. One study combined already developed commercial platforms and developed its own scenarios using various technologies [97].

**Table 1:** Categories of technology applied in simulation-based learning to enhance critical thinking in the included studies (N=96).

Category of applied technology	Studies, n (%)
CBS^a^ [52,53,55,59,67,76,78,83,85,86,89,90,93,96,97,99,101,104,105,108,109,111,114,117-125,127,128,130-133,136-140,143]	44 (46)
HPS^b^ [51,57,60,62-66,68-73,80-82,84,87,91,94,100,107,141,145,146]	26 (27)
VR^c^ or IVR^d^ [75,77,95,98,103,110,126]	7 (7)
Other—combination of technologies, technologies not consistent with the first 3 categories, or unclear description of technology [54,56,58,61,74,79,88,102,106,110,112,113,115,116,129,134,135,142,144]	19 (20)

^a^CBS: computer-based simulation.

^b^HPS: human-patient simulator.

^c^VR: virtual reality.

^d^IVR: immersive virtual reality.

### Use of Technology in SBL Activities to Enhance Critical Thinking (RQ 2)

#### Technology Integration Into SBL

HPS technology was integrated into SBL mainly by nursing students playing out active roles in real-world clinical scenarios, with HPSs representing patients. SBL was typically described as being built around clinical scenarios, with nursing students as nurses caring for the patient by assessing and taking necessary measures in the scenario [51, 62-64, 68-71, 81, 82, 91, 94, 96, 100, 107, 141, 144, 146]. SBL using telehealth technology in the studies was built around clinical telehealth scenarios [79,135,142]. The digital technology used in TES, CBS, and VR or IVR was usually built around clinical cases and scenarios in which nursing students took on the role of a nurse caring for a patient. Nursing students applied their knowledge and skills to assess, solve problems, and make clinical decisions [53, 59, 67, 78, 83, 85-90, 93, 96, 101-104, 106, 109, 112, 114, 115, 118, 120-122, 124, 125, 128, 130, 131, 133, 134, 136, 140, 143]. Several studies (34/61, 56%) incorporated scenario design and game mechanics aimed at developing clinical reasoning or clinical judgment and critical thinking skills [61, 75, 77, 85-90, 92, 96,97,99, 101-104, 109, 111, 112, 114, 116, 117, 119, 122, 124-126, 130-132, 137, 139, 144]. Some studies (12/61, 20%) stated that they used a narrative approach, constructing patient stories that evolved over time following a cause-and-effect structure to demonstrate the consequences of decisions and actions, thereby contributing to the development of critical thinking [77,85,88-90,105,111,117,122,128,131,143].

#### Synchronous and Asynchronous Learning Activities

The studies used technology in SBL to promote critical thinking through both synchronous and asynchronous learning activities. The learning activities in SBL that used an HPS were predominantly conducted synchronously, featuring scenarios in real time that aimed to enhance critical thinking and the clinical reasoning process through experiential learning [51, 56, 57, 60, 62-66, 68-73, 81, 82, 84, 87,91, 94, 100, 107, 113, 116, 135, 141, 144, 145]. These learning activities were conducted in groups ranging from pairs to larger numbers of nursing students. Asynchronous learning activities were often designed using TESs in SBL intended for self-directed, individual learning, providing more flexibility and independence for nursing students [52, 53, 55, 59, 61, 67, 78, 83, 85, 88, 92, 99, 104, 108, 113, 114, 116-122, 125, 127-129, 131-134, 140, 144]. This approach also gave nursing students the possibility of repetition and repetitive learning [61,99,109,127,134,144]. A third approach to using technology in SBL was to combine asynchronous and synchronous learning activities. The most common technologies applied for the combined approach were CBS and VR or IVR [77, 86, 90, 93, 96-99, 101-103, 105, 110-112, 115, 124, 126, 130, 136-139, 144], with SBL most commonly conducted as a combination of individual performance in CBS, VR, or IVR followed by group reflections and debriefing facilitated by trained faculty. One study reported conducting all parts of SBL using CBS synchronously on the web via a videoconferencing platform [89].

Other combinations included videotaped vignettes of a scenario combined with individual performance testing [54], an HPS in combination with multimedia in prerecorded simulated assessments, offering eighter synchronous or asynchronous debriefing for online students using a learning management system [58], and guided workshops of CBS content before asynchronous individual learning [109]. The studies that used a combined approach were predominantly published after 2019 as 50% (8/16) of the included studies in the first updated search (2021-2023) and 46% (13/28) in the second updated search (2023-2024) reported using this approach compared to 6% (5/52) of the studies in the initial search (1986-2021). Of the 96 included studies, 6 (6%) did not give sufficient descriptions of the learning activities [75,76,80,95,123,146].

#### Feedback for Developing Critical Thinking Skills

The feedback described in studies that used HPS technology in SBL was provided in 2 ways: feedback provided by the HPSs giving human patient responses to actions taken during the scenario, including both physical reactions and verbal interactions, and feedback provided by educators or peers during the debriefing phase of SBL (21/96, 22%) [51,56,57,60,62-66,68-74,81,84,87,91,94,100,107,112,135,141]. In the SBL modality that used FaceTime and audiovisual equipment for telehealth scenarios, feedback was also provided during debriefing by trained facilitators [79,135,142]. A total of 3% (3/96) of the studies reported using video recordings during debriefing to evaluate, reflect, and provide feedback on student performance [62,72,100]. One study reported feedback provided through asynchronous discussion within a learning management system [58]. TESs using CBS and VR or IVR technology provided other and additional feedback models compared to SBL using HPS technology, with both audio and visual automated responses. Some TESs provided feedback in real time as the game unfolded or cumulatively at the end of the scenario or gaming session [59, 88, 89, 96, 109, 110, 112, 114, 115, 121, 122, 125, 127-129, 131]. Feedback in both CBS and VR or IVR was also delivered indirectly through patients’ responses to actions and communication with a cause-and-effect approach that demonstrated the consequences of decisions and actions taken [75,103,110,117,129,131,143]. Feedback was given in diverse forms, such as written messages or verbal feedback from a patient, via facilitators or peers during the SBL session [90,115,127-129], quizzes within the CBS [102], and pop-up messages or summaries on execution compared to expected nursing performance at the end of the VR, IVR, or CBS session [52,55,95,102,103,108,109,120,136]. CBSs could provide feedback tailored to debriefing purposes [93,117,143]. In some studies (9/61, 15%), TESs provided students with a performance score, typically in the form of points or a percentage reflecting the accuracy of their actions when the game was completed [53,61,78,86,108,127,128,136]. Several studies (10/61, 16%) using TESs combined feedback from CBSs or IVR with face-to-face synchronous debriefing and group discussions [86,89,90,101-104,110,114,124].

### Enablers and Barriers of the Applied Technology in SBL to Enhance Critical Thinking (RQ 3)

#### Overview

From the inductive data analyses, seven distinct themes emerged: (1) affinity for and availability of technology, (2) realism, (3) accessibility, (4) engagement and motivation, (5) validation, (6) return on investment, and (7) enhanced critical thinking through SBL using technology. The studies explored aspects of developing critical thinking skills through learning in SBL using technological modalities such as HPSs, CBSs, VR or IVR, and others. An overview of the dimensions of the D&M IS Success Model [48], the identified themes, their respective subthemes, and examples of both barriers and enablers is provided in Table 2.

**Table 2:** 

Dimension of the D&M IS Success Model, main theme, and subthemes				Examples of measures for IS success enablers		Examples of measures for IS success barriers
**System quality—ease of learning, ease of use (usability), intuitiveness, flexibility, reliability, response times, availability, and desirable characteristics**						
	**Affinity for and availability of technology**					
		Understanding technology	Well-structured and logical design [87,90,131].		Challenges in understanding computer or accessory context [85,108,121] Outdated software [138]	
		Understanding game features	Easy to navigate [131]		Challenges in navigating the game setting [92,108,123,138]	
		Flexibility	Possibility of repetition [58,75,97,103,109,110,112,125] Native language [120] Independent of time and space [103,109,121,131]		Foreign language [124,138]	
**Information quality—relevance, completeness, accuracy, conciseness, personalization, understandability, currency, timeliness, and security**						
	**Realism**					
		Real-life scenarios	Provides all aspects of the patient and context, giving a realistic experience [58, 75, 84, 88, 90, 102, 103, 106, 108, 112, 122, 124-126, 131, 138, 142]		Low flexibility for changing a situation (compromised reality) [58,81,110] Situations lacking resemblance to real life [82,113,123]	
		Physiological realism	Replication of real human physiological features [75,81,107,131,138]		Limited human features of the patient presented by technology [81,82,87,107]	
		Theory-to-practice transition	Being able to visualize patient status and the effect of measures [75,81,107,108,131]		Technology with low and intermediate fidelity [81,82] Context perceived as not relevant [123]	
**Service quality—the quality of the support that system users receive from the service providers; this could entail IT support as well as other types of user support and empathy from staff**						
	**Accessibility**					
		Technical accessibility	Technology functioning as expected [142]		Technical problems that cause interruptions [92,110,120,124] Technical issues such as internet access and connection [90,120,138]	
**Use (behavior) and intention to use (attitude)—amount, frequency, use patterns, appropriateness, and purpose of use; attitude toward using and reusing the system**						
	**Engagement and motivation**					
		Preferred learning styles	Visual learning [106-108,120,122,125] Autonomy in learning [75,77,97,106,130,131,138] Being challenged [77,81,84,92,107,108,122,126,132,136]		Perceived as not sufficient for “hands-on” learning [110,120,123] Limited variability [113,121]	
		Physiological and psychological experiences	Safe learning environment [87,88,109,110,112,122,124,126] Immersion [92,97,103,108,110,112,126] Sparked curiosity [108,113,126] Activation of thoughts and feelings [92,108,122,131]		Negative physiological reactions [89,106,110] Confusion [82] Feeling of incompetence and frustration and low sense of mastery and self-efficacy [82,108]	
		Experienced use	Time to become familiar with features of the technology [79,142] Proper training and instructions [79,110,142] Appropriate taxonomical level [97,107]		Limited time to become familiar with technology [103,107,138] Viewing the learning tool negatively [108,123] Content not adapted to the taxonomical level [89,107,123] Inappropriate time frame allotted to tasks [123]	
	**Validation**					
		Need for feedback	Feedback provided on the actions taken [79,82,97,103,107,109,112,138] Feedback enables engagement [102,107,109,125,138] Provision of examples and explanation of how to perform patient care and why [53,79,125,138]		No feedback offered [58] Insufficient feedback offered [81,85]	
**Satisfaction—overall opinions about the system**						
	**Return on investment**					
		Student investment	No information available		No value for money [123]	
**Net benefits—impacts on individual users, groups, organizations, industries, and nations**						
	**Enhanced critical thinking through SBL** ^a^ **using technology**					
		Meta-cognition	Reflecting on one’s own actions and thinking [58,75,81,83,84,92,97,109,123,124,126] Self-reflection and self-evaluation [58,75,83,97,126,131]		Insufficient debriefing provided [107,112]	

^a^SBL: simulation-based learning.

#### System Quality: Affinity and Availability

This theme pertains primarily to TESs, in which students interact with technology such as CBS and VR or IVR in self-directed, individual learning activities. Well-structured and logical technological platforms with easy navigation were reported as perceived enablers for achieving outcomes in SBL [87,90,131]. Challenges in understanding technological and game features [85,108,121], outdated software [138], and challenges navigating the technological platform [92,108,123,138] were reported as barriers because they limited options and influenced the interaction and the flow of the simulation [92,108,138]. Disorientation and difficulties obtaining information were viewed as time-consuming and hindering to nursing students’ learning process, which caused frustration and negative experiences [92,108,123,138]. Flexibility encompassed the use of TESs individually and regardless of time and space [109,121,131] and the possibility of repetition [58,75,97,103,109,110,112,125], which was perceived positively in terms of motivation to use the applied technology and improve performance [58,75,97]. Interacting with TESs in the students’ native language enhanced the availability of learning situations [120]. Options in a foreign language and lack of multiple language options were reported perceived barriers that made learning less available to nursing students [124,138].

#### Information Quality: Realism

Technology that provided all aspects of patient context with a realistic experience was reported as likely to enable learning and the development of critical thinking [58, 75, 84, 88, 90, 102, 103, 106, 108, 112, 122, 125, 126, 131, 138, 142]. Realistic replication of the human physiological features of the patient [75,81,107,131,138], realistic changes in patients’ conditions, and being able to visually inspect the patient’s status and see the effect of measures [75,81,107,108,131] were reported as increasing perceived realism; theory-to-practice transition; and, hence, nursing students’ development of critical thinking skills. Low flexibility for changes in the scenario [58,81,110], limited resemblance to real-life situations [82,113,123], and failure to replicate realistic human features of the patient [81,82,87,107] were factors of technology that were reported to compromise realism in SBL [58,81,82,107,110,123] and cause confusion for nursing students.

#### Service Quality: Accessibility

When technology functions as expected, it may enhance critical thinking by making learning opportunities accessible [142]. Technical problems caused interruptions that interfered with learning and the smooth development of the scenario in SBL and were reported as a barrier to the development of critical thinking skills by negatively influencing the accessibility of the learning opportunity [82,120]. Another barrier reported, especially with TESs conducted individually, was technical issues related to internet access and connection [120,138].

#### Use and Intention to Use: Engagement, Motivation, and Validation

The preferred learning style may influence nursing students’ engagement and motivation for interacting with technology in SBL and achieve critical thinking outcomes. Nursing students reported that the visual elements of the technology enabled the development of critical thinking through increased engagement from visual learners [106-108,120,122,125]. The perceived autonomy of self-directed learning in TESs, with the opportunity to try out behaviors and apply knowledge and skills without being supervised [75,77,97,106,130,131,138], as well as being challenged on skills and knowledge [77, 81, 84, 92, 97, 107, 108, 126, 132, 138], increased nursing students engagement and motivation. Some nursing students perceived that SBL using HPSs, CBSs, or VR or IVR did not constitute hands-on learning and, hence, was not appropriate for gaining enhanced critical thinking skills outside the clinical environment [110,120,123]. Across several studies (13/42, 31%), nursing students reported that TESs provided a safe learning environment [87,88,109,110,112,122,124,126] where a high level of immersion [92,97,103,108,110,112,126], sparked curiosity [108,113,126], and the activation of thoughts and feelings [92,108,122,131] enabled involvement and engagement and facilitated the development of critical thinking for nursing students. Limited variability and opportunity to explore inhibited motivation for some nursing students [113,121]. Negative physiological reactions from immersive technology, such as dizziness and headaches, influenced nursing students’ engagement and motivation [89,106,110]. Emotions such as confusion, frustration, feelings of incompetency, and a low sense of mastery related to technology led to decreased engagement and loss of motivation and, hence, critical thinking development [82,108].

Experienced use of technology in SBL influenced engagement and motivation. Time to become familiar with features of the technology [79,142], proper training [79,110,142], and appropriate taxonomical level [97,107] were factors associated with enhanced motivation to engage in learning reported by nursing students. Failure to become familiar with the technology [103,107,138], content not adapted to the appropriate taxonomical level [89,107,123], and an inappropriate time frame for conducting a task [123] resulted in decreased engagement and motivation for nursing students, leading to a negative perception of the technology [108,123].

Validation and feedback with provision of examples and explanations of patient care [53,79,125,138] and feedback on actions taken [79,82,97,103,107,109,112,138] were perceived as valuable for nursing students in SBL, enabling critical thinking development [79,82,97,105,107,138]. Feedback enabled motivation and engagement [102,107,109,125,138]. Insufficient or absence of feedback led to uncertainties regarding nursing students’ own performance and the accuracy of their problem-solving, ultimately influencing the learning experience and development of critical thinking skills [58,81,85].

#### Satisfaction: Return on Investment

Chircop et al [123] found that nursing students were not satisfied with the CBS program they were required to buy and that several expressed that the CBS was not perceived as providing value for the money spent.

#### Net Benefits: SBL Using Technology

Nursing students reported that they perceived that SBL using technology can enhance critical thinking development through opportunities for meta-cognition and engagement in reflection [58,75,77,97,123,138]. Nursing students expressed a perceived value of opportunities to think about their own actions and thinking [58,75,81,83,84,92,97,109,123,124,126], engage in self-reflection and self-evaluation, and consider the benefits of receiving and giving feedback in debriefing sessions with peers and faculty [58,75,83,97,126,131]. Insufficient or no debriefing could decrease nursing students’ opportunities for meta-cognition and development of critical thinking [107,112]

## Discussion

### Principal Findings

This scoping review aimed to systematically map studies on the use of SBL supported by technology to enhance critical thinking in nursing students. We identified 4 main categories of applied technology in SBL to enhance critical thinking as well as a shift across time in the use of the technology applied: from HPSs to CBSs. The technology applied in SBL to enhance critical thinking skills was closely connected to pedagogical considerations and educational approaches. This technology was applied in asynchronous and synchronous learning approaches. Learning approaches and use of technology in SBL have developed over time and trend toward a more blended or combined educational approach after 2019. Our main findings on RQ 3 were related to the information quality and use and intention to use dimensions of the D&M IS Success Model, with themes associated with realism as well as engagement and motivation as prominent findings. The included studies were diverse in origin, which indicates that the topic of interest is relevant worldwide. However, 44% (42/96) of the included studies originated in the United States. This poses the question of whether the use of technology in SBL to enhance critical thinking is more emphasized in the United States or whether this is an expression of a geographical bias in conducting research on the topic. Another concern is about the global applicability. Although a significant proportion of the studies in this review (42/96, 44%) were from the United States, it is important to consider whether and how the findings can be transferred to other educational contexts. Educational systems vary significantly worldwide, and factors such as cultural differences, resource availability, and teaching methods can impact the effectiveness of SBL supported by technology. For instance, in countries with limited resources, access to advanced technology may be a challenge. This may affect the implementation and outcomes of SBL. In addition, cultural differences in learning styles and teaching methods may require adaptations to ensure that SBL methods are effective [147]. It is also crucial to consider how different health care systems and nursing education requirements can influence the transferability of the findings. For example, in countries with different nursing education standards, adaptations may be necessary to ensure that SBL is relevant and effective [148].

### Range of Technology

The included studies spanned a 38-year time frame, necessitating consideration of the significant technological development during this period. The technologies applied in SBL must be evaluated within the context of the studies’ time frames as they might be outdated by present standards. From a historical perspective, the findings showed a shift in the technology applied in SBL to enhance critical thinking from HPS to TES modalities, such as CBS and VR or IVR. From 2018 to 2023, the predominant technology was CBS, with even more studies conducted using VR or IVR technology than HPSs. This showcases the evolving landscape of educational technology for SBL and active learning in nursing education. Although there is an increased use of health care and telehealth technology in SBL for future registered nurses [149], our findings suggest that there is limited research on the use of telehealth technology, which is in line with the results of a study by Giuffrida et al [28]. The lack of studies on the use of telehealth in SBL to enhance critical thinking is striking considering the global emphasis on technology and technological advancements as well as the impact of the COVID-19 pandemic and its consequences. The limited number of studies regarding telehealth technology in SBL could be due to publication delay [150], and more research could emerge in the near future.

The descriptions of the applied technology varied across the studies. To consider the validity and generalizability or transferability of the findings in relation to technology as well as compare findings across studies, detailed descriptions of the technology are necessary [151,152]. Our review suggests that studies incorporating a digital technology into TESs generally provided a richer description of the technology involved than those that used HPSs. This may be due to the technology being more novel and complex and researchers acknowledging the importance of providing this information.

### Use of Technology in SBL Activities to Enhance Critical Thinking

The use of technology in terms of learning design and teaching strategies in SBL to enhance critical thinking varied across the included studies. Synchronous learning was predominant in SBL that used HPSs, featuring real-time scenarios with physical presence. Conversely, digital technology such as TES leaned toward asynchronous activities, fostering self-directed learning with flexibility for learners. Wong et al [153] argue that developing skills to optimize self-directed learning is essential, with senior nursing students potentially being more adapted to mastering this skill compared to their junior counterparts, who may require more support, guidance, and validation. The need for validation, especially in self-directed learning within SBL, indicates the importance of aligning technological tools with educational objectives and providing comprehensive support for nursing students. As highlighted by the International Nursing Association of Clinical Simulation and Learning [154], there is a need to align tools or modalities in SBL with specific learning outcomes in the design phase of SBL. According to Nadelson et al [155], several nursing programs are looking to provide nursing students with more opportunities for online SBL, and the authors indicate that one important pedagogical choice is whether this should be provided as a synchronous or asynchronous learning activity. Research suggests that both formats yield similar outcomes, with TES offering efficiency through standardized content and the possibility of individualized training [156]. The potential for asynchronous, individualized training and multiple iterations as offered by TES could make these modalities resource efficient due to lower staff costs [25,26,157], with time freed for supportive teaching activities [158]. Hence, pedagogical considerations and cost efficiency should be determined before the choice of technological modality.

The evolution of teaching strategies was evident in the included studies published after 2019, with an increased number of studies that reported combining asynchronous and synchronous aspects in learning activities. According to Kalanlar [149], findings across 30 international nursing education programs reveal that 81% transitioned from traditional teaching to online classes during the pandemic, and of these, 61% conducted SBL using TES, 12% conducted SBL using HPSs, and 10% conducted SBL using telehealth. However, nursing students have expressed low satisfaction with excessive asynchronous activities and pointed out quality differences compared to tactile learning, thus highlighting the challenges of online SBL [149,159]. In a national postpandemic survey in the United States, nursing students reported that the virtual environment and online SBL were inadequate to prepare them for clinical reasoning in direct patient care [160]. The shift to online nursing education accelerated by the COVID-19 pandemic posed challenges to both educators and students, and this may have prompted the shift to a more blended teaching approach [161]. This evolution tends toward combined approaches in teaching critical thinking skills using technology in SBL, typically through individual asynchronous scenarios conducted virtually with TESs followed by synchronous debriefing. This may signal a shift toward a more balanced and comprehensive teaching strategy that considers aspects of social constructivism in learning, in which meaningful interactions influence learning positively [162]. The trend of applying a blended learning approach may represent a recognition that technology is not yet capable of replacing an experienced facilitator in guiding proper reflection processes. Debriefing, integral to SBL, facilitates reflection and learning, with feedback playing a crucial role in developing critical thinking skills [163,164]. Our review identified several methods of receiving feedback for the development of critical thinking through SBL using different technological modalities and tools. Consistent with a systematic review of VR in nursing education [15], we found that TESs offered more diverse feedback models and debriefing practices compared with HPSs, such as scoring systems and real-time feedback on actions taken and communication. This prompts exploration of the factors influencing the choice of educational strategies applied in nursing education to achieve the desired outcome and the optimal technology and design for SBL for effective critical thinking development.

### Enablers and Barriers of the Applied Technology in SBL to Enhance Critical Thinking

An evident finding is the crucial role of realism, including real-life scenarios and the replication of human features in simulated patients, for nursing students’ learning enhancement. Our findings showed that nursing students’ perceptions of realistic patients, with both consistent human and physiological features and contexts, enable learning and enhance critical thinking skills. This could link to nursing students’ experience of learning opportunities’ relevance in SBL, connecting them to real nursing experiences. Nursing students value realistic features in the SBL environment to perform effectively. The term *relational realism* refers to the degree of realism experienced when interacting with HPSs in a simulated clinical setting [165]. Learning activities using an HPS in SBL may fail if there is no perceived relational realism, leading to learners being reluctant to interact and engage. In designing SBL, educators need to consider several aspects of reality, such as physiological, psychological, and conceptual realism, as well as technological simulation modalities and tools [21]. Furthermore, experiences of immersion were reported by nursing students to enhance realism and, hence, learning and critical thinking skill development through engagement, motivation, and emotional activation. The literature supports that immersion and interactivity are key to virtual technology learning experiences [166]. The effectiveness of VR or IVR technologies relies on the users’ sense of presence [167]. Motivation theories highlight the impact of emotions on students’ engagement in learning activities, suggesting the importance of realism factors, as noted by Dubovi [168]. These insights emphasize the importance of creating SBL environments that mirror real-life patients and contexts closely for nurturing critical thinking skills in nursing education.

Our findings highlight the pivotal role of engagement, motivation, and personalized learning styles, emphasizing the need for adaptive and learner-centered approaches in designing and implementing technology in SBL through different modalities and tools. A systematic review on the use of VR among nursing students and registered nurses identified 2 disadvantages with the virtual world: a lack of realism and technical issues [169]. Our scoping review suggests that technical issues and problems could compromise realism and be barriers to learning and the development of critical thinking skills as these issues limit the availability of learning opportunities in the learning environment. The absence of realism may diminish immersion, consequently reducing engagement and the achievement of learning outcomes. Our results emphasize the importance of nursing students’ technological proficiency in effectively interacting with technology. Harerimana and Mtshali [170] found that students’ computer skills influenced their perception of the effectiveness of teaching and learning using technology. Consequently, using teaching and learning strategies to facilitate students’ development of computer proficiency and technological literacy may enhance learning using technology in other areas, such as the development of critical thinking skills [46]. A lack of technological proficiency can be a major source of frustration for nursing students, potentially leading to a negative perception of technology [169] and decreasing their motivation and engagement [170]. Studies have identified poorly structured and designed digital learning interventions and technical problems as major causes of negative outcomes when introducing digital learning interventions in higher education [170,171]. They emphasize that technical difficulties diminish the value of the learning experience, decrease motivation, and contribute to feelings of inequality among students. This is particularly crucial in synchronous learning situations, in which interaction is essential [171]. Coupled with providing comprehensive technical support, ensuring technological functionality and quality is crucial for motivating and engaging nursing students in technology-based learning activities to enhance their critical thinking skills.

### Theoretical Foundation for Pedagogy and Outcome Evaluation

The variety in theoretical foundations and the diverse outcome measures identified in our review pose a challenge regarding the comparison of research outcomes. More than half (56/96, 58%) of the studies in our scoping review incorporated an underlying theory or conceptual framework to inform their interventions. Establishing a scientific theoretical foundation for future studies may secure more robust research outcomes in the field of SBL with the support of technology to enhance critical thinking. Bauce et al [172] highlight that theory is needed in research to support pedagogy and emphasize that theory has served to underpin pedagogy and provide a foundation for outcome evaluation. The use of scientific theories in SBL research is important in formulating precise RQs, guiding study design and analysis, and contributing to a cumulative research program. It is crucial to differentiate between scientific theories, which are explanatory and descriptive, and a conceptual framework, which represents some aspects of reality [173]. Conceptual frameworks have a different scientific meaning from that of a scientific theory, and thus, the studies conducted based on them are different in nature. Varpio et al [174] underscore the need for a standardized understanding of terms such as *theory*, *theoretical framework*, and *conceptual framework* within health profession education and research. This is essential for enhancing the clarity and rigor of research reporting, subsequently fostering a more robust theoretical foundation for future studies in the field of SBL supported by technology to augment critical thinking skills. Furthermore, there was diversity in the validated instruments and self-developed questionnaires used across the included studies to measure outcomes. Adib-Hajbaghery and Sharifi [29] question the use of general measurement instruments to assess the outcome of critical thinking when evaluating effects in relation to specialized nursing interventions. This underscores the necessity for standardization and consensus on global research practices, facilitating more meaningful cross-study analyses and enabling the development of comprehensive reviews and meta-analyses.

### Future Research Directions

The extensive body of research, comprising 96 studies included in this scoping review, highlights the potential for future systematic reviews and meta-analyses of both quantitative and qualitative scientific studies on SBL using technology to enhance critical thinking. This would provide deeper insights into the effectiveness of SBL strategies and identify best practices for integrating technology to foster critical thinking development. Furthermore, there is a need for consistency and standardization in the use of scientific theory to enhance the clarity and rigor of research reporting and establish a robust theoretical foundation for future studies in the field of SBL supported by technology to augment nursing students’ critical thinking skills.

Future research should investigate SBL in relation to different learning styles, variations in SBL outcomes among nursing students at different stages of their education, generational differences in technology use, content and design of SBL, and the choice of technology applied to achieve the outcome of enhanced critical thinking. Studies should also explore factors influencing educators’ choice of technology and design for SBL in nursing education to achieve outcomes and the enhancement of critical thinking skills in nursing students. Despite the increasing emphasis on health care technology and telehealth, there is limited research on such technology use in SBL in nursing education to enhance nursing students’ critical thinking skills. This calls for further research to inform nursing education on the pedagogical aspects of SBL using health care technology generally and telehealth technology specifically to enhance nursing students’ critical thinking skills. Furthermore, studies comparing and evaluating the impact of different technologies on higher-order thinking outcomes may provide valuable insights that could inform nursing education.

### Strengths and Limitations

The strengths of this scoping review were that our scoping review protocol was peer reviewed and published, and we used an acknowledged methodological framework and reported the review according to PRISMA-ScR checklist [38,39] to enhance transparency (Multimedia Appendix 4). A comprehensive search strategy was developed in collaboration with an experienced research librarian. The inclusion of various concepts often considered synonymous with critical thinking that share several similar factors was important due to the vague and diverse definitions of critical thinking. However, the fact that the studies may have used these concepts in ways that are not conceptually equivalent must be considered. Our inclusion criteria were limited to specified languages. Consequently, we may have introduced selection bias. Other limitations may be the dominant population of undergraduate students in the included studies, which might limit transferability to other contexts and populations. Furthermore, the dominance of included studies originating in the United States should be considered when interpreting the findings. However, we believe that, by including studies from 20 different countries, this reflects SBL with technology to enhance critical thinking in nursing education as a universal theme and the review exhibits global trends in nursing education and the use of technology in SBL to enhance critical thinking. Therefore, an understanding of each country’s educational system is imperative in interpreting the findings. In addition, the variability in theoretical foundations and outcome measures across the studies may have hindered cross-study comparisons in this review. Finally, the findings need to be interpreted with caution due to the diversity in research contexts, technology used, and SBL interventions in the included studies and because the methodological quality of the included studies was not appraised and the data were organized and not synthesized.

### Conclusions

This scoping review highlights a global relevance through a range of diverse studies. Over time, there was a noticeable shift from HPSs to TESs such as CBS and VR or IVR, emphasizing the importance of detailed descriptions of the applied technology to ensure validity and comparability across studies. After 2018, a trend toward blended educational approaches combining asynchronous and synchronous learning activities emerged. The feedback provided to nursing students in SBL to enhance critical thinking varied in delivery, with technology-driven feedback tailored to learning objectives playing a pivotal role in enhancing critical thinking. This highlights the need for educators to carefully select and tailor technology-based tools and programs to deliver feedback aligned with learning objectives and outcomes of critical thinking development. The establishment of robust theoretical foundations of research and standardized research practices will strengthen the evidence obtained from the research conducted. Realism, user proficiency, and the perceived quality of the technology significantly influence engagement, motivation, and the development of critical thinking in nursing students. This review provides valuable insights for educators, designers, and technology developers to enhance the effectiveness of technologically supported SBL in nursing education. Our findings can inform evidence-based practices, guide the design of effective educational interventions, and contribute to the ongoing discourse on the integration of technology into SBL in nursing education.

## Appendix

Multimedia Appendix 1 Deviations from the published protocol.

Multimedia Appendix 2 Search strategy in Ovid MEDLINE.

Multimedia Appendix 3 Characteristics of the included studies.

Multimedia Appendix 4 PRISMA-ScR (Preferred Reporting Items for Systematic Reviews and Meta-Analyses extension for Scoping Reviews) checklist.

## Abbreviations

CBS: computer-based simulation
D&M: DeLone and McLean
HPS: human-patient simulator
IS: information system
IVR: immersive virtual reality
PRISMA-ScR: Preferred Reporting Items for Systematic Reviews and Meta-Analyses extension for Scoping Reviews
RQ: research question
SBL: simulation-based learning
TES: technology-enhanced simulation
VR: virtual reality
VS: virtual simulation

## Data Availability

All data generated or analyzed during this study are included in this published article (and its supplementary information files).
