# Supplementary material for: Simulation-Based Learning Supported by Technology to Enhance Critical Thinking in Nursing Students: Scoping Review
Source: J Med Internet Res. 2025 Feb 18;27:e58744. doi: 10.2196/58744 (PMC11888118; doi:10.2196/58744)
Supplement: Multimedia Appendix 1 [file jmir_v27i1e58744_app1.docx]

## Multimedia appendix 1. Deviations from the published protocol

| **Planned Approach described in the protocol** | **Type, description of and justification for the**  **deviation from protocol** |
| --- | --- |
| **Methods**  The aim of the scoping review is to systematically map **research** on the use of simulation-based learning supported by technology to enhance critical thinking in nursing students. | Type of deviation: clarification/change  The formulation is changed to: This scoping review aimed to systematically map **studies** on the use of SBL supported by technology to enhance critical thinking in NSs. |
| Methods  The protocol states that the database search will be updated 3 months prior to submission. | Type of deviation: clarification/change  Because of the extensive amount of data, the research team needed more time than anticipated to analyze the dataset. The updated search was conducted March 17th, 2023, a year before submission. This is stated in the method section. |
| Data analysis  HVS, SAS, MTS, and AAGN will analyze the results from the included papers and will use an inductive approach to organize the results thematically, a method previously used in scoping reviews. | Type of deviation: clarification/change & addition  Results was analyzed accordingly by first and last author; HVS and AAGN. There was a deviation regarding RQ3 as a result of the analyzing process and the data retrieved: HVS & AAGN analyzed the qualitative data with an inductive and deductive approach. This is described in the methods section and results displayed in results section. |
| Methods  We planned to present a frequency table showing which papers appear in which thematic groupings will be created. | Type of deviation: clarification/change  Considering the number of included studies (n=67), a frequency table showing the respective papers in the deductive analysis was developed, as we found this to be the best approach to present an overview of the main qualitative analysis. Presented in the results section (Table ?) |
| Methods  We anticipated that the scoping review will be completed by May 2023. | Type of deviation: clarification/change  Due to the number of included papers and large dataset the Scoping review was delayed and not completed before submission in March 2024 |
| Methods  Further, the same pairs of authors will independently assess whether the full-text papers meet the inclusion criteria. | Type of deviation: clarification/change  Due to changes in composition of the research team, there were some changes in the pairs conducting the full text papers. The pair of authors were also slightly altered for the study selection and data charting process for the updated search. |
| Methods  We proposed that a standardized data collection form will be developed in Microsoft Word for data extraction from the included papers, including *authors, year, country, aim, sample, design, technology, simulation procedures, scenario design, and results related to the research question.* | Type of deviation: addition and clarification/change  We developed a more substantial collection form in Microsoft excel *authors, year, country, aim, sample, design, applied theory/ theoretical foundation for CT, Evaluation tools, technology, Description of technology, simulation procedures, intervention approach and scenario design, results related to the research question, and results not related to research questions (but important/interesting findings)*. The addition of the categories: *Applied theory/ theoretical foundation for CT, Evaluation tools and results not related to research questions (but important/interesting findings)* were included after the pilot trial of the form. There was a need for the additional categories to fully answer the research questions. The category scenario design was renamed intervention approach and scenario design to give more comprehensive data of the interventions in studies. |
