# Supplementary material for: Simulation-Based Learning Supported by Technology to Enhance Critical Thinking in Nursing Students: Scoping Review
Source: J Med Internet Res. 2025 Feb 18;27:e58744. doi: 10.2196/58744 (PMC11888118; doi:10.2196/58744)
Supplement: Multimedia Appendix 2 [file jmir_v27i1e58744_app2.docx]

## Multimedia Appendix 2. Search strategy in Ovid MEDLINE

All searches were last conducted 13th of November 2024

Ovid MEDLINE All

| 1 | simulat*.tw,kf. |
| --- | --- |
| 2 | sbl.tw,kf. |
| 3 | (manikin* or mannequin*).tw,kf. |
| 4 | ("high-fidelity" or "low-fidelity").tw,kf. |
| 5 | (skill* adj2 training*).tw,kf. |
| 6 | (Standardi#ed adj3 Patient*).tw,kf. |
| 7 | (Anatomic* adj2 Model*).tw,kf. |
| 8 | ("Role* play*" or roleplay*).tw,kf. |
| 9 | (skill* adj2 (Lab or labs or laborator*)).tw,kf. |
| 10 | (Learning adj2 (Lab or labs or laborator*)).tw,kf. |
| 11 | ((Practical or practice) adj1 (Lab or labs or laborator*)).tw,kf. |
| 12 | exp Simulation Training/ |
| 13 | Models, Anatomic/ |
| 14 | Role Playing/ |
| 15 | Manikins/ |
| 16 | or/1-15 |
| 17 | Computer Simulation/ |
| 18 | augmented reality/ |
| 19 | virtual reality/ |
| 20 | vr.tw,kf. |
| 21 | ((virtual* or augmented) adj2 (realit* or environment*1 or room* or patient* or simulation*)).tw,kf. |
| 22 | ((web or online or computer*) adj3 simulation*).tw,kf. |
| 23 | ("e-simulation*" or esimulation*).tw,kf. |
| 24 | 17 or 18 or 19 or 20 or 21 or 22 or 23 |
| 25 | exp Telemedicine/ |
| 26 | Telecommunications/ or exp Telemetry/ or Wireless Technology/ or exp Videoconferencing/ or User-Computer Interface/ or Computer assisted instruction/ or Internet-Based Intervention/ or Audiovisual Aids/ |
| 27 | Mobile Applications/ or exp Telephone/ |
| 28 | computers/ or microcomputers/ or computers, handheld/ or smartphone/ or minicomputers/ or exp Video Games/ |
| 29 | (((wearable or wireless) adj2 (technolog* or electronic)) or (digital adj2 medicine) or (technolog* adj2 health) or ((mobile or internet or electronic* or robot*) adj2 (consultation* or application*1))).tw,kf. |
| 30 | (telecommunicat* or tele-communicat* or teleconferenc* or tele-conferenc*or app or apps or app-based or mobile-based or "Short Message Service*" or sms or textmessag* or text-messag* or texting or videoconferenc* or video-conferenc* or webconferenc* or web-conferenc* or webcast* or web-cast* or webinar* or web-application* or web-based-application*).tw,kf. |
| 31 | (phone*1 or telephon* or smartphone* or smart-phone* or cellphone* or cell-phone* or mobilephone* or mobile-phone* or "personal digital assistant*" or palmpilot* or palm-pilot* or smarthome* or smart-home* or tablet*1).tw,kf. |
| 32 | (telemedicin* or tele-medicin* or telehealth* or tele-health* or telecare* or tele-care* or telecari* or tele-cari* or emedic* or e-medic* or ehealth* or e-health* or mhealth* or m-health* or ehomecare* or e-homecare* or e-home-care* or teleconsultation* or tele-consultation* or videoconsultation* or video-consultation* or telenurs* or tele-nurs* or teletherap* or tele-therap* or telerehab* or tele-rehab* or erehab* or e-rehab* or telemonitor* or tele-monitor*).tw,kf. |
| 33 | high-tech*.tw,kf. |
| 34 | (game* or gaming or gamification or gamify or videogame* or "video game*").tw,kf. |
| 35 | ((computer or multimedia or digital* or online) adj5 (program* or education* or environment* or learn* or interface* or instruction* or based or platform* or device* or app or apps or application*)).tw,kf. |
| 36 | technolog*.tw,kf. |
| 37 | (touchscreen* or "touch screen*").tw,kf. |
| 38 | ((portable or handheld or hand-held) adj1 computer*).tw,kf. |
| 39 | or/25-38 |
| 40 | ((nurse or nursing or nurses) adj4 (student* or education or undergrad* or under-grad* or bachelor* or baccalaur* or preregistration or pre-registration or post* or practitioner* or school* or college*)).tw,kf. |
| 41 | exp Education, Nursing/ |
| 42 | Students, Nursing/ |
| 43 | or/40-42 |
| 44 | 16 and 39 |
| 45 | 24 or 44 |
| 46 | 43 and 45 |
| 47 | limit 46 to (danish or english or norwegian or portuguese or spanish or swedish) |

Ovid Embase

| 1 | simulat*.tw,kw. |
| --- | --- |
| 2 | sbl.tw,kw. |
| 3 | (manikin* or mannequin*).tw,kw. |
| 4 | ("high-fidelity" or "low-fidelity").tw,kw. |
| 5 | (skill* adj2 training*).tw,kw. |
| 6 | (Standardi#ed adj3 Patient*).tw,kw. |
| 7 | (Anatomic* adj2 Model*).tw,kw. |
| 8 | ("role* play*" or roleplay*).tw,kw. |
| 9 | ((Learning or skill*) adj2 (Lab or labs or laborator*)).tw,kw. |
| 10 | ((Practical or practice) adj1 (Lab or labs or laborator*)).tw,kw. |
| 11 | simulation/ or disease simulation/ or high-fidelity simulation/ or patient simulation/ or vignette/ |
| 12 | exp anatomic model/ |
| 13 | role playing/ |
| 14 | or/1-13 |
| 15 | exp computer simulation/ |
| 16 | vr.tw,kw. |
| 17 | ((virtual* or augmented) adj2 (realit* or environment*1 or room* or patient* or simulation*)).tw,kw. |
| 18 | ((web or online or computer*) adj3 simulation*).tw,kw. |
| 19 | ("e-simulation*" or esimulation*).tw,kw. |
| 20 | or/15-19 |
| 21 | exp telehealth/ or exp telemetry/ or telecommunication/ or wireless communication/ or videoconferencing/ or web-based intervention/ |
| 22 | telephone/ or mobile phone/ or smartphone/ or exp mobile application/ |
| 23 | computer interface/ or human machine interface/ or audiovisual aid/ or audiovisual equipment/ |
| 24 | computer/ or computer system/ or computer terminal/ or digital computer/ or microcomputer/ or minicomputer/ or personal computer/ or personal digital assistant/ |
| 25 | video game/ |
| 26 | (((wearable or wireless) adj2 (technolog* or electronic)) or (digital adj2 medicine) or (technolog* adj2 health) or ((mobile or internet or electronic* or robot*) adj2 (consultation* or application*1))).tw,kw. |
| 27 | (telecommunicat* or tele-communicat* or teleconferenc* or tele-conferenc*or app or apps or app-based or mobile-based or "Short Message Service*" or sms or textmessag* or text-messag* or texting or videoconferenc* or video-conferenc* or webconferenc* or web-conferenc* or webcast* or web-cast* or webinar* or web-application* or web-based-application*).tw,kw. |
| 28 | (phone*1 or telephon* or smartphone* or smart-phone* or cellphone* or cell-phone* or mobilephone* or mobile-phone* or "personal digital assistant*" or palmpilot* or palm-pilot* or smarthome* or smart-home* or tablet*1).tw,kw. |
| 29 | (telemedicin* or tele-medicin* or telehealth* or tele-health* or telecare* or tele-care* or telecari* or tele-cari* or emedic* or e-medic* or ehealth* or e-health* or mhealth* or m-health* or ehomecare* or e-homecare* or e-home-care* or teleconsultation* or tele-consultation* or videoconsultation* or video-consultation* or telenurs* or tele-nurs* or teletherap* or tele-therap* or telerehab* or tele-rehab* or erehab* or e-rehab* or telemonitor* or tele-monitor*).tw,kw. |
| 30 | high-tech*.tw,kw. |
| 31 | (game* or gaming or gamification or gamify or videogame* or "video game*").tw,kw. |
| 32 | ((computer or multimedia or digital* or online) adj5 (program* or education* or environment* or learn* or interface* or instruction* or based or platform* or device* or app or apps or application*)).tw,kw. |
| 33 | technolog*.tw,kw. |
| 34 | (touchscreen* or "touch screen*").tw,kw. |
| 35 | ((portable or handheld or hand-held) adj1 computer*).tw,kw. |
| 36 | or/21-35 |
| 37 | ((nurse or nursing or nurses) adj4 (student* or education or undergrad* or under-grad* or bachelor* or baccalaur* or preregistration or pre-registration or post* or practitioner* or school* or college*)).tw,kw. |
| 38 | exp nursing student/ |
| 39 | exp nursing education/ |
| 40 | or/37-39 |
| 41 | 14 and 36 |
| 42 | 20 or 41 |
| 43 | 40 and 42 |
| 44 | limit 43 to (danish or english or norwegian or portuguese or spanish or swedish) |

Ovid APA PsycInfo

| 1 | simulat*.tw. |
| --- | --- |
| 2 | sbl.tw. |
| 3 | (manikin* or mannequin*).tw. |
| 4 | ("high-fidelity" or "low-fidelity").tw. |
| 5 | (skill* adj2 training*).tw. |
| 6 | (Standardi#ed adj3 Patient*).tw. |
| 7 | (Anatomic* adj2 Model*).tw. |
| 8 | ("role* play*" or roleplay*).tw. |
| 9 | ((Learning or skill*) adj2 (Lab or labs or laborator*)).tw. |
| 10 | ((Practical or practice) adj1 (Lab or labs or laborator*)).tw. |
| 11 | simulation/ |
| 12 | Simulation Games/ |
| 13 | role playing/ |
| 14 | role playing games/ |
| 15 | or/1-14 |
| 16 | computer simulation/ or exp virtual reality/ |
| 17 | vr.tw. |
| 18 | ((virtual* or augmented) adj2 (realit* or environment*1 or room* or patient* or simulation*)).tw. |
| 19 | ((web or online or computer*) adj3 simulation*).tw. |
| 20 | ("e-simulation*" or esimulation*).tw. |
| 21 | 16 or 17 or 18 or 19 or 20 |
| 22 | exp electronic health services/ |
| 23 | computer applications/ or computer assisted diagnosis/ or computer assisted instruction/ or computer assisted therapy/ or computer software/ or electronic collaboration/ or exp electronic learning/ or groupware/ or hypermedia/ or mobile applications/ |
| 24 | computer assisted instruction/ or computer supported collaborative learning/ or exp electronic learning/ or intelligent tutoring systems/ or decision support systems/ or computational reinforcement learning/ |
| 25 | digital gaming/ or computer games/ or digital game-based learning/ |
| 26 | exp Computers/ |
| 27 | exp wireless technologies/ or digital technology/ or electronic communication/ or exp mobile phones/ or laptop computers/ or exp mobile devices/ or streaming technology/ |
| 28 | (((wearable or wireless) adj2 (technolog* or electronic)) or (digital adj2 medicine) or (technolog* adj2 health) or ((mobile or internet or electronic* or robot*) adj2 (consultation* or application*1))).tw. |
| 29 | (telecommunicat* or tele-communicat* or teleconferenc* or tele-conferenc*or app or apps or app-based or mobile-based or "Short Message Service*" or sms or textmessag* or text-messag* or texting or videoconferenc* or video-conferenc* or webconferenc* or web-conferenc* or webcast* or web-cast* or webinar* or web-application* or web-based-application*).tw. |
| 30 | (phone*1 or telephon* or smartphone* or smart-phone* or cellphone* or cell-phone* or mobilephone* or mobile-phone* or "personal digital assistant*" or palmpilot* or palm-pilot* or smarthome* or smart-home* or tablet*1).tw. |
| 31 | (telemedicin* or tele-medicin* or telehealth* or tele-health* or telecare* or tele-care* or telecari* or tele-cari* or emedic* or e-medic* or e-health* or ehealth* or mhealth* or m-health* or ehomecare* or e-homecare* or e-home-care* or teleconsultation* or tele-consultation* or videoconsultation* or video-consultation* or telenurs* or tele-nurs* or teletherap* or tele-therap* or telerehab* or tele-rehab* or erehab* or e-rehab* or telemonitor* or tele-monitor*).tw. |
| 32 | high-tech*.tw. |
| 33 | (game* or gaming or gamification or gamify or videogame* or "video game*").tw. |
| 34 | ((computer or multimedia or digital* or online) adj5 (program* or education* or environment* or learn* or interface* or instruction* or based or platform* or device* or app or apps or application*)).tw. |
| 35 | technolog*.tw. |
| 36 | (touchscreen* or "touch screen*").tw. |
| 37 | ((portable or handheld or hand-held) adj1 computer*).tw. |
| 38 | or/22-37 |
| 39 | ((nurse or nursing or nurses) adj4 (student* or education or undergrad* or under-grad* or bachelor* or baccalaur* or preregistration or pre-registration or post* or practitioner* or school* or college*)).tw. |
| 40 | nursing education/ |
| 41 | nursing students/ |
| 42 | 39 or 40 or 41 |
| 43 | 15 and 38 |
| 44 | 21 or 43 |
| 45 | 42 and 44 |
| 46 | limit 45 to (danish or english or norwegian or portuguese or spanish or swedish) |

LILACS

(simulation OR "anatomic models" OR "anatomic model" OR manikin OR manikins OR mannequin OR mannequins OR "role* play*" OR roleplay*) AND (nurs*) AND (technolog* OR game* OR gaming OR videogame* OR “video game” OR gamify OR gamification OR vr OR virtual* OR augmented OR computer* OR telehealth* OR telemedicine* OR telenurs* OR smartphone* OR "smart phone" OR "smart phones" OR "cell phone" OR "cell phones*" OR cellphone OR cellphones OR tablet OR tablets) AND ( db:("LILACS" OR "BDENF" OR "IBECS")) AND ( db:("LILACS" OR "BDENF" OR "IBECS"))

EBSCO CINAHL Complete

| **#** | **Query** | **Limiters/Expanders** | **Last Run Via** |
| --- | --- | --- | --- |
| S1 | (MH "Simulations") OR (MH "Patient Simulation") OR (MH "Vignettes") OR (MH "Role Playing") | Search modes - Boolean/Phrase | Interface - EBSCOhost Research Databases |
|  |  |  | Search Screen - Advanced Search |
|  |  |  | Database - CINAHL Complete |
| S2 | (MH "Models, Anatomic+") | Search modes - Boolean/Phrase | Interface - EBSCOhost Research Databases |
|  |  |  | Search Screen - Advanced Search |
|  |  |  | Database - CINAHL Complete |
| S3 | (MH "Learning Laboratories") | Search modes - Boolean/Phrase | Interface - EBSCOhost Research Databases |
|  |  |  | Search Screen - Advanced Search |
|  |  |  | Database - CINAHL Complete |
| S4 | (MH "Learning Environment, Clinical") | Search modes - Boolean/Phrase | Interface - EBSCOhost Research Databases |
|  |  |  | Search Screen - Advanced Search |
|  |  |  | Database - CINAHL Complete |
| S5 | TI (simulat* OR SBL) OR AB (simulat* OR SBL) | Search modes - Boolean/Phrase | Interface - EBSCOhost Research Databases |
|  |  |  | Search Screen - Advanced Search |
|  |  |  | Database - CINAHL Complete |
| S6 | TI (manikin* or mannequin*) OR AB (manikin* or mannequin*) | Search modes - Boolean/Phrase | Interface - EBSCOhost Research Databases |
|  |  |  | Search Screen - Advanced Search |
|  |  |  | Database - CINAHL Complete |
| S7 | TI ("high-fidelity" or "low-fidelity") OR AB ("high-fidelity" or "low-fidelity") | Search modes - Boolean/Phrase | Interface - EBSCOhost Research Databases |
|  |  |  | Search Screen - Advanced Search |
|  |  |  | Database - CINAHL Complete |
| S8 | TI (skill* N1 training) OR AB (skill* N1 training) | Search modes - Boolean/Phrase | Interface - EBSCOhost Research Databases |
|  |  |  | Search Screen - Advanced Search |
|  |  |  | Database - CINAHL Complete |
| S9 | TI (Standardi#ed N2 Patient*) OR AB (Standardi#ed N2 Patient*) | Search modes - Boolean/Phrase | Interface - EBSCOhost Research Databases |
|  |  |  | Search Screen - Advanced Search |
|  |  |  | Database - CINAHL Complete |
| S10 | TI (Anatomic* n1 Model*) OR AB (Anatomic* n1 Model*) | Search modes - Boolean/Phrase | Interface - EBSCOhost Research Databases |
|  |  |  | Search Screen - Advanced Search |
|  |  |  | Database - CINAHL Complete |
| S11 | TI (("Role* play*") OR roleplay*) OR AB (("Role* play*") OR roleplay*) | Search modes - Boolean/Phrase | Interface - EBSCOhost Research Databases |
|  |  |  | Search Screen - Advanced Search |
|  |  |  | Database - CINAHL Complete |
| S12 | TI ((Learning or skill*) n1 (Lab or labs or laborator*)) OR AB ((Learning or skill*) n1 (Lab or labs or laborator*)) | Search modes - Boolean/Phrase | Interface - EBSCOhost Research Databases |
|  |  |  | Search Screen - Advanced Search |
|  |  |  | Database - CINAHL Complete |
| S13 | TI ((Practical OR practice) n0 (Lab or labs or laborator*)) OR AB ((Practical OR practice) n0 (Lab or labs or laborator*)) | Search modes - Boolean/Phrase | Interface - EBSCOhost Research Databases |
|  |  |  | Search Screen - Advanced Search |
|  |  |  | Database - CINAHL Complete |
| S14 | S1 OR S2 OR S3 OR S4 OR S5 OR S6 OR S7 OR S8 OR S9 OR S10 OR S11 OR S12 OR S13 | Search modes - Boolean/Phrase | Interface - EBSCOhost Research Databases |
|  |  |  | Search Screen - Advanced Search |
|  |  |  | Database - CINAHL Complete |
| S15 | (MH "Computer Simulation+") | Search modes - Boolean/Phrase | Interface - EBSCOhost Research Databases |
|  |  |  | Search Screen - Advanced Search |
|  |  |  | Database - CINAHL Complete |
| S16 | (MH "Virtual Reality+") | Search modes - Boolean/Phrase | Interface - EBSCOhost Research Databases |
|  |  |  | Search Screen - Advanced Search |
|  |  |  | Database - CINAHL Complete |
| S17 | TI VR OR AB VR | Search modes - Boolean/Phrase | Interface - EBSCOhost Research Databases |
|  |  |  | Search Screen - Advanced Search |
|  |  |  | Database - CINAHL Complete |
| S18 | TI ((virtual* or augmented) N1 (realit* or environment or environments or room* or patient* or simulation*)) OR AB ((virtual* or augmented) N1 (realit* or environment or environments or room* or patient* or simulation*)) | Search modes - Boolean/Phrase | Interface - EBSCOhost Research Databases |
|  |  |  | Search Screen - Advanced Search |
|  |  |  | Database - CINAHL Complete |
| S19 | TI ((web or online or computer*) N2 simulation*) OR AB ((web or online or computer*) N2 simulation*) | Search modes - Boolean/Phrase | Interface - EBSCOhost Research Databases |
|  |  |  | Search Screen - Advanced Search |
|  |  |  | Database - CINAHL Complete |
| S20 | TI ("e-simulation*" or esimulation*) OR AB ("e-simulation*" or esimulation*) | Search modes - Boolean/Phrase | Interface - EBSCOhost Research Databases |
|  |  |  | Search Screen - Advanced Search |
|  |  |  | Database - CINAHL Complete |
| S21 | S15 OR S16 OR S17 OR S18 OR S19 OR S20 | Search modes - Boolean/Phrase | Interface - EBSCOhost Research Databases |
|  |  |  | Search Screen - Advanced Search |
|  |  |  | Database - CINAHL Complete |
| S22 | (MH "Telehealth+") | Search modes - Boolean/Phrase | Interface - EBSCOhost Research Databases |
|  |  |  | Search Screen - Advanced Search |
|  |  |  | Database - CINAHL Complete |
| S23 | (MH "Telecommunications") OR (MH "Videoconferencing+") OR (MH "Internet-Based Intervention") OR (MH "Wireless Communications") OR (MH "Telephone") OR (MH "Cellular Phone") OR (MH "Smartphone") OR (MH "Telecommuting") OR (MH "Teleconferencing") | Search modes - Boolean/Phrase | Interface - EBSCOhost Research Databases |
|  |  |  | Search Screen - Advanced Search |
|  |  |  | Database - CINAHL Complete |
| S24 | (MH "Mobile Applications") | Search modes - Boolean/Phrase | Interface - EBSCOhost Research Databases |
|  |  |  | Search Screen - Advanced Search |
|  |  |  | Database - CINAHL Complete |
| S25 | (MH "Computer Types+") | Search modes - Boolean/Phrase | Interface - EBSCOhost Research Databases |
|  |  |  | Search Screen - Advanced Search |
|  |  |  | Database - CINAHL Complete |
| S26 | (MH "Video Games+") | Search modes - Boolean/Phrase | Interface - EBSCOhost Research Databases |
|  |  |  | Search Screen - Advanced Search |
|  |  |  | Database - CINAHL Complete |
| S27 | TI (((wearable or wireless) N1 (technolog* or electronic)) or (digital N1 medicine) or (technolog* N1 health) or ((mobile or internet or electronic* or robot*) N1 (consultation* or application or applications))) OR AB (((wearable or wireless) N1 (technolog* or electronic)) or (digital N1 medicine) or (technolog* N1 health) or ((mobile or internet or electronic* or robot*) N1 (consultation* or application or applications))) | Search modes - Boolean/Phrase | Interface - EBSCOhost Research Databases |
|  |  |  | Search Screen - Advanced Search |
|  |  |  | Database - CINAHL Complete |
| S28 | TI (telecommunicat* or tele-communicat* or teleconferenc* or tele-conferenc* or teleconsultation* or tele-consultation* or videoconsultation* or video-consultation* or telenurs* or tele-nurs* or telemonitor* or tele-monitor* or teletherap* or tele-therap* or telerehab* or tele-rehab* or erehab* or e-rehab* or app or apps or app-based or mobile-based or "Short Message Service*" or sms or textmessag* or text-messag* or texting or videoconferenc* or video-conferenc* or webconferenc* or web-conferenc* or webcast* or web-cast* or webinar* or web-application* or web-based-application*) OR AB (telecommunicat* or tele-communicat* or teleconferenc* or tele-conferenc* or teleconsultation* or tele-consultation* or videoconsultation* or video-consultation* or telenurs* or tele-nurs* or telemonitor* or tele-monitor* or teletherap* or tele-therap* or telerehab* or tele-rehab* or erehab* or e-rehab* or app or apps or app-based or mobile-based or "Short Message Service*" or sms or textmessag* or text-messag* or texting or videoconferenc* or video-conferenc* or webconferenc* or web-conferenc* or webcast* or web-cast* or webinar* or web-application* or web-based-application*) | Search modes - Boolean/Phrase | Interface - EBSCOhost Research Databases |
|  |  |  | Search Screen - Advanced Search |
|  |  |  | Database - CINAHL Complete |
| S29 | TI (phone* or phones or telephon* or smartphone* or smart-phone* or cellphone* or cell-phone* or mobilephone* or mobile-phone* or "personal digital assistant*" or palmpilot* or palm-pilot* or smarthome* or smart-home* or tablet* or tablets) OR AB (phone* or phones or telephon* or smartphone* or smart-phone* or cellphone* or cell-phone* or mobilephone* or mobile-phone* or "personal digital assistant*" or palmpilot* or palm-pilot* or smarthome* or smart-home* or tablet* or tablets) | Search modes - Boolean/Phrase | Interface - EBSCOhost Research Databases |
|  |  |  | Search Screen - Advanced Search |
|  |  |  | Database - CINAHL Complete |
| S30 | TI (telemedicin* or tele-medicin* or telehealth* or tele-health* or telecare* or tele-care* or telecari* or tele-cari* or emedic* or e-medic* or e-health* or ehealth* or mhealth* or m-health* or ehomecare* or e-homecare* or e-home-care*) OR AB (telemedicin* or tele-medicin* or telehealth* or tele-health* or telecare* or tele-care* or telecari* or tele-cari* or emedic* or e-medic* or e-health* or ehealth* or mhealth* or m-health* or ehomecare* or e-homecare* or e-home-care*) | Search modes - Boolean/Phrase | Interface - EBSCOhost Research Databases |
|  |  |  | Search Screen - Advanced Search |
|  |  |  | Database - CINAHL Complete |
| S31 | TI (high-tech*) OR AB (high-tech*) | Search modes - Boolean/Phrase | Interface - EBSCOhost Research Databases |
|  |  |  | Search Screen - Advanced Search |
|  |  |  | Database - CINAHL Complete |
| S32 | TI (game* or gaming or gamification or gamify or videogame* or "video game*") OR AB (game* or gaming or gamification or gamify or videogame* or "video game*") | Search modes - Boolean/Phrase | Interface - EBSCOhost Research Databases |
|  |  |  | Search Screen - Advanced Search |
|  |  |  | Database - CINAHL Complete |
| S33 | TI ((computer or multimedia or digital* or online) N4 (program* or education* or environment* or learn* or interface* or instruction* or based or platform* or device* or app or apps or application*)) OR AB ((computer or multimedia or digital* or online) N4 (program* or education* or environment* or learn* or interface* or instruction* or based or platform* or device* or app or apps or application*)) | Search modes - Boolean/Phrase | Interface - EBSCOhost Research Databases |
|  |  |  | Search Screen - Advanced Search |
|  |  |  | Database - CINAHL Complete |
| S34 | TI technolog* OR AB technolog* | Search modes - Boolean/Phrase | Interface - EBSCOhost Research Databases |
|  |  |  | Search Screen - Advanced Search |
|  |  |  | Database - CINAHL Complete |
| S35 | TI (touchscreen* or "touch screen*") OR AB (touchscreen* or "touch screen*") | Search modes - Boolean/Phrase | Interface - EBSCOhost Research Databases |
|  |  |  | Search Screen - Advanced Search |
|  |  |  | Database - CINAHL Complete |
| S36 | TI ((portable or handheld or hand-held) N0 computer*) OR AB ((portable or handheld or hand-held) N0 computer*) | Search modes - Boolean/Phrase | Interface - EBSCOhost Research Databases |
|  |  |  | Search Screen - Advanced Search |
|  |  |  | Database - CINAHL Complete |
| S37 | S22 OR S23 OR S24 OR S25 OR S26 OR S27 OR S28 OR S29 OR S30 OR S31 OR S32 OR S33 OR S34 OR S35 OR S36 | Search modes - Boolean/Phrase | Interface - EBSCOhost Research Databases |
|  |  |  | Search Screen - Advanced Search |
|  |  |  | Database - CINAHL Complete |
| S38 | TI ((nurse or nursing or nurses) N3 (student* or education or undergrad* or under-grad* or bachelor* or baccalaur* or preregistration or pre-registration or post* or practitioner* or school* or college*)) OR AB ((nurse or nursing or nurses) N3 (student* or education or undergrad* or under-grad* or bachelor* or baccalaur* or preregistration or pre-registration or post* or practitioner* or school* or college*)) | Search modes - Boolean/Phrase | Interface - EBSCOhost Research Databases |
|  |  |  | Search Screen - Advanced Search |
|  |  |  | Database - CINAHL Complete |
| S39 | (MH "Education, Nursing+") OR (MH "Education, Midwifery") | Search modes - Boolean/Phrase | Interface - EBSCOhost Research Databases |
|  |  |  | Search Screen - Advanced Search |
|  |  |  | Database - CINAHL Complete |
| S40 | (MH "Students, Nursing+") | Search modes - Boolean/Phrase | Interface - EBSCOhost Research Databases |
|  |  |  | Search Screen - Advanced Search |
|  |  |  | Database - CINAHL Complete |
| S41 | S38 OR S39 OR S40 | Search modes - Boolean/Phrase | Interface - EBSCOhost Research Databases |
|  |  |  | Search Screen - Advanced Search |
|  |  |  | Database - CINAHL Complete |
| S42 | S14 AND S37 | Search modes - Boolean/Phrase | Interface - EBSCOhost Research Databases |
|  |  |  | Search Screen - Advanced Search |
|  |  |  | Database - CINAHL Complete |
| S43 | S21 OR S42 | Search modes - Boolean/Phrase | Interface - EBSCOhost Research Databases |
|  |  |  | Search Screen - Advanced Search |
|  |  |  | Database - CINAHL Complete |
| S44 | S41 AND S43 | Search modes - Boolean/Phrase | Interface - EBSCOhost Research Databases |
|  |  |  | Search Screen - Advanced Search |
|  |  |  | Database - CINAHL Complete |
| S45 | S41 AND S43 | Limiters - Language: Danish, English, Norwegian, Portuguese, Spanish, Swedish | Interface - EBSCOhost Research Databases |
|  |  | Search modes - Boolean/Phrase | Search Screen - Advanced Search |
|  |  |  | Database - CINAHL Complete |

EBSCO ERIC

| **#** | **Query** | **Limiters/Expanders** | **Last Run Via** |
| --- | --- | --- | --- |
| S1 | DE "Simulation" OR DE "Role Playing" OR DE "Simulated Environment" OR DE "Vignettes" | Search modes - Boolean/Phrase | Interface - EBSCOhost Research Databases |
|  |  |  | Search Screen - Advanced Search |
|  |  |  | Database - ERIC |
| S2 | (DE "Learning Laboratories") | Search modes - Boolean/Phrase | Interface - EBSCOhost Research Databases |
|  |  |  | Search Screen - Advanced Search |
|  |  |  | Database - ERIC |
| S3 | (DE "Educational Games") | Search modes - Boolean/Phrase | Interface - EBSCOhost Research Databases |
|  |  |  | Search Screen - Advanced Search |
|  |  |  | Database - ERIC |
| S4 | TI (simulat* OR SBL) OR AB (simulat* OR SBL) | Search modes - Boolean/Phrase | Interface - EBSCOhost Research Databases |
|  |  |  | Search Screen - Advanced Search |
|  |  |  | Database - ERIC |
| S5 | TI (manikin* or mannequin*) OR AB (manikin* or mannequin*) | Search modes - Boolean/Phrase | Interface - EBSCOhost Research Databases |
|  |  |  | Search Screen - Advanced Search |
|  |  |  | Database - ERIC |
| S6 | TI ("high-fidelity" or "low-fidelity") OR AB ("high-fidelity" or "low-fidelity") | Search modes - Boolean/Phrase | Interface - EBSCOhost Research Databases |
|  |  |  | Search Screen - Advanced Search |
|  |  |  | Database - ERIC |
| S7 | TI (skill* N1 training) OR AB (skill* N1 training) | Search modes - Boolean/Phrase | Interface - EBSCOhost Research Databases |
|  |  |  | Search Screen - Advanced Search |
|  |  |  | Database - ERIC |
| S8 | TI (Standardi#ed N2 Patient*) OR AB (Standardi#ed N2 Patient*) | Search modes - Boolean/Phrase | Interface - EBSCOhost Research Databases |
|  |  |  | Search Screen - Advanced Search |
|  |  |  | Database - ERIC |
| S9 | TI (("Role* play*") OR roleplay*) OR AB (("Role* play*") OR roleplay*) | Search modes - Boolean/Phrase | Interface - EBSCOhost Research Databases |
|  |  |  | Search Screen - Advanced Search |
|  |  |  | Database - ERIC |
| S10 | TI (Anatomic* n1 Model*) OR AB (Anatomic* n1 Model*) | Search modes - Boolean/Phrase | Interface - EBSCOhost Research Databases |
|  |  |  | Search Screen - Advanced Search |
|  |  |  | Database - ERIC |
| S11 | TI ((Learning or skill*) n1 (Lab or labs or laborator*)) OR AB ((Learning or skill*) n1 (Lab or labs or laborator*)) | Search modes - Boolean/Phrase | Interface - EBSCOhost Research Databases |
|  |  |  | Search Screen - Advanced Search |
|  |  |  | Database - ERIC |
| S12 | TI ((Practical OR practice) n0 (Lab or labs or laborator*)) OR AB ((Practical OR practice) n0 (Lab or labs or laborator*)) | Search modes - Boolean/Phrase | Interface - EBSCOhost Research Databases |
|  |  |  | Search Screen - Advanced Search |
|  |  |  | Database - ERIC |
| S13 | S1 OR S2 OR S3 OR S4 OR S5 OR S6 OR S7 OR S8 OR S9 OR S10 OR S11 OR S12 | Search modes - Boolean/Phrase | Interface - EBSCOhost Research Databases |
|  |  |  | Search Screen - Advanced Search |
|  |  |  | Database - ERIC |
| S14 | DE "Computer Simulation" | Search modes - Boolean/Phrase | Interface - EBSCOhost Research Databases |
|  |  |  | Search Screen - Advanced Search |
|  |  |  | Database - ERIC |
| S15 | TI VR OR AB VR | Search modes - Boolean/Phrase | Interface - EBSCOhost Research Databases |
|  |  |  | Search Screen - Advanced Search |
|  |  |  | Database - ERIC |
| S16 | TI ((web or online or computer*) N2 simulation*) OR AB ((web or online or computer*) N2 simulation*) | Search modes - Boolean/Phrase | Interface - EBSCOhost Research Databases |
|  |  |  | Search Screen - Advanced Search |
|  |  |  | Database - ERIC |
| S17 | TI ("e-simulation*" or esimulation*) OR AB ("e-simulation*" or esimulation*) | Search modes - Boolean/Phrase | Interface - EBSCOhost Research Databases |
|  |  |  | Search Screen - Advanced Search |
|  |  |  | Database - ERIC |
| S18 | TI ((virtual* or augmented) N1 (realit* or environment or environments or room* or patient* or simulation*)) OR AB ((virtual* or augmented) N1 (realit* or environment or environments or room* or patient* or simulation*)) | Search modes - Boolean/Phrase | Interface - EBSCOhost Research Databases |
|  |  |  | Search Screen - Advanced Search |
|  |  |  | Database - ERIC |
| S19 | S14 OR S15 OR S16 OR S17 or S18 | Search modes - Boolean/Phrase | Interface - EBSCOhost Research Databases |
|  |  |  | Search Screen - Advanced Search |
|  |  |  | Database - ERIC |
| S20 | TI (telemedicin* or tele-medicin* or telehealth* or tele-health* or telecare* or tele-care* or telecari* or tele-cari* or emedic* or e-medic* or e-health* or ehealth* or mhealth* or m-health* or ehomecare* or e-homecare* or e-home-care*) OR AB (telemedicin* or tele-medicin* or telehealth* or tele-health* or telecare* or tele-care* or telecari* or tele-cari* or emedic* or e-medic* or e-health* or ehealth* or mhealth* or m-health* or ehomecare* or e-homecare* or e-home-care*) | Search modes - Boolean/Phrase | Interface - EBSCOhost Research Databases |
|  |  |  | Search Screen - Advanced Search |
|  |  |  | Database - ERIC |
| S21 | TI (high-tech*) OR AB (high-tech*) | Search modes - Boolean/Phrase | Interface - EBSCOhost Research Databases |
|  |  |  | Search Screen - Advanced Search |
|  |  |  | Database - ERIC |
| S22 | (DE "Telecommunications" OR DE "Computer Mediated Communication" OR DE "Teleconferencing" OR DE "Videoconferencing" OR DE "Teleworking") | Search modes - Boolean/Phrase | Interface - EBSCOhost Research Databases |
|  |  |  | Search Screen - Advanced Search |
|  |  |  | Database - ERIC |
| S23 | (DE "Electronic Learning" OR DE "Computers" OR DE "Computer Software" OR DE "Computer Assisted Instruction" OR DE "Computer Interfaces" OR DE "Laptop Computers") | Search modes - Boolean/Phrase | Interface - EBSCOhost Research Databases |
|  |  |  | Search Screen - Advanced Search |
|  |  |  | Database - ERIC |
| S24 | DE "Video Games" | Search modes - Boolean/Phrase | Interface - EBSCOhost Research Databases |
|  |  |  | Search Screen - Advanced Search |
|  |  |  | Database - ERIC |
| S25 | TI (((wearable or wireless) N1 (technolog* or electronic)) or (digital N1 medicine) or (technolog* N1 health) or ((mobile or internet or electronic* or robot*) N1 (consultation* or application or applications))) OR AB (((wearable or wireless) N1 (technolog* or electronic)) or (digital N1 medicine) or (technolog* N1 health) or ((mobile or internet or electronic* or robot*) N1 (consultation* or application* or applications))) | Search modes - Boolean/Phrase | Interface - EBSCOhost Research Databases |
|  |  |  | Search Screen - Advanced Search |
|  |  |  | Database - ERIC |
| S26 | TI (telecommunicat* or tele-communicat* or teleconferenc* or tele-conferenc* or teleconsultation* or tele-consultation* or videoconsultation* or video-consultation* or telenurs* or tele-nurs* or telemonitor* or tele-monitor* or teletherap* or tele-therap* or telerehab* or tele-rehab* or erehab* or e-rehab* or app or apps or app-based or mobile-based or "Short Message Service*" or sms or textmessag* or text-messag* or texting or videoconferenc* or video-conferenc* or webconferenc* or web-conferenc* or webcast* or web-cast* or webinar* or web-application* or web-based-application*) OR AB (telecommunicat* or tele-communicat* or teleconferenc* or tele-conferenc* or teleconsultation* or tele-consultation* or videoconsultation* or video-consultation* or telenurs* or tele-nurs* or telemonitor* or tele-monitor* or teletherap* or tele-therap* or telerehab* or tele-rehab* or erehab* or e-rehab* or app or apps or app-based or mobile-based or "Short Message Service*" or sms or textmessag* or text-messag* or texting or videoconferenc* or video-conferenc* or webconferenc* or web-conferenc* or webcast* or web-cast* or webinar* or web-application* or web-based-application*) | Search modes - Boolean/Phrase | Interface - EBSCOhost Research Databases |
|  |  |  | Search Screen - Advanced Search |
|  |  |  | Database - ERIC |
| S27 | TI (phone or phones or telephon* or smartphone* or smart-phone* or cellphone* or cell-phone* or mobilephone* or mobile-phone* or "personal digital assistant*" or palmpilot* or palm-pilot* or smarthome* or smart-home* or tablet or tablets) OR AB (phone or phones or telephon* or smartphone* or smart-phone* or cellphone* or cell-phone* or mobilephone* or mobile-phone* or "personal digital assistant*" or palmpilot* or palm-pilot* or smarthome* or smart-home* or tablet or tablets) | Search modes - Boolean/Phrase | Interface - EBSCOhost Research Databases |
|  |  |  | Search Screen - Advanced Search |
|  |  |  | Database - ERIC |
| S28 | TI (game* or gaming or gamification or gamify or videogame* or "video game*") OR AB (game* or gaming or gamification or gamify or videogame* or "video game*") | Search modes - Boolean/Phrase | Interface - EBSCOhost Research Databases |
|  |  |  | Search Screen - Advanced Search |
|  |  |  | Database - ERIC |
| S29 | TI ((computer or multimedia or digital* or online) N4 (program* or education* or environment* or learn* or interface* or instruction* or based or platform* or device* or app or apps or application*)) OR AB ((computer or multimedia or digital* or online) N4 (program* or education* or environment* or learn* or interface* or instruction* or based or platform* or device* or app or apps or application*)) | Search modes - Boolean/Phrase | Interface - EBSCOhost Research Databases |
|  |  |  | Search Screen - Advanced Search |
|  |  |  | Database - ERIC |
| S30 | TI technolog* OR AB technolog* | Search modes - Boolean/Phrase | Interface - EBSCOhost Research Databases |
|  |  |  | Search Screen - Advanced Search |
|  |  |  | Database - ERIC |
| S31 | TI (touchscreen* or "touch screen*") OR AB (touchscreen* or "touch screen*") | Search modes - Boolean/Phrase | Interface - EBSCOhost Research Databases |
|  |  |  | Search Screen - Advanced Search |
|  |  |  | Database - ERIC |
| S32 | TI ((portable or handheld or hand-held) N0 computer*) OR AB ((portable or handheld or hand-held) N0 computer*) | Search modes - Boolean/Phrase | Interface - EBSCOhost Research Databases |
|  |  |  | Search Screen - Advanced Search |
|  |  |  | Database - ERIC |
| S33 | S20 OR S21 OR S22 OR S23 OR S24 OR S25 OR S26 OR S27 OR S28 OR S29 OR S30 OR S31 OR S32 | Search modes - Boolean/Phrase | Interface - EBSCOhost Research Databases |
|  |  |  | Search Screen - Advanced Search |
|  |  |  | Database - ERIC |
| S34 | TI ((nurse or nursing or nurses) N3 (student* or education or undergrad* or under-grad* or bachelor* or baccalaur* or preregistration or pre-registration or post* or practitioner* or school* or college*)) OR AB ((nurse or nursing or nurses) N3 (student* or education or undergrad* or under-grad* or bachelor* or baccalaur* or preregistration or pre-registration or post* or practitioner* or school* or college*)) | Search modes - Boolean/Phrase | Interface - EBSCOhost Research Databases |
|  |  |  | Search Screen - Advanced Search |
|  |  |  | Database - ERIC |
| S35 | DE "Nursing Education" OR DE "Nursing Students" | Search modes - Boolean/Phrase | Interface - EBSCOhost Research Databases |
|  |  |  | Search Screen - Advanced Search |
|  |  |  | Database - ERIC |
| S36 | S34 OR S35 | Search modes - Boolean/Phrase | Interface - EBSCOhost Research Databases |
|  |  |  | Search Screen - Advanced Search |
|  |  |  | Database - ERIC |
| S37 | S13 AND S33 | Search modes - Boolean/Phrase | Interface - EBSCOhost Research Databases |
|  |  |  | Search Screen - Advanced Search |
|  |  |  | Database - ERIC |
| S38 | S19 OR S37 | Search modes - Boolean/Phrase | Interface - EBSCOhost Research Databases |
|  |  |  | Search Screen - Advanced Search |
|  |  |  | Database - ERIC |
| S39 | S36 AND S38 | Search modes - Boolean/Phrase | Interface - EBSCOhost Research Databases |
|  |  |  | Search Screen - Advanced Search |
|  |  |  | Database - ERIC |
| S40 | S36 AND S38 | Limiters - Language: Danish, English, Portuguese, Spanish; Castilian, Swedish | Interface - EBSCOhost Research Databases |
|  |  | Search modes - Boolean/Phrase | Search Screen - Advanced Search |
|  |  |  | Database - ERIC |

ISI Web of Science

| # 31 | #30 AND #29 |
| --- | --- |
|  | *Indexes=SCI-EXPANDED, SSCI, A&HCI, ESCI Timespan=All years* |
|  |  |
| # 30 | TS=((nurse or nursing or nurses) NEAR/3 (student* or education or undergrad* or under-grad* or bachelor* or baccalaur* or preregistration or pre-registration or post* or practitioner* or school* or college*) ) |
|  | *Indexes=SCI-EXPANDED, SSCI, A&HCI, ESCI Timespan=All years* |
|  |  |
| # 29 | #28 OR #16 |
|  | *Indexes=SCI-EXPANDED, SSCI, A&HCI, ESCI Timespan=All years* |
|  |  |
| # 28 | #27 AND #12 |
|  | *Indexes=SCI-EXPANDED, SSCI, A&HCI, ESCI Timespan=All years* |
|  |  |
| # 27 | #26 OR #25 OR #24 OR #23 OR #22 OR #21 OR #20 OR #19 OR #18 OR #17 |
|  | *Indexes=SCI-EXPANDED, SSCI, A&HCI, ESCI Timespan=All years* |
|  |  |
| # 26 | TS=((portable or handheld or hand-held) NEAR/0 computer*) |
|  | *Indexes=SCI-EXPANDED, SSCI, A&HCI, ESCI Timespan=All years* |
|  |  |
| # 25 | TS=(touchscreen* or "touch screen*") |
|  | *Indexes=SCI-EXPANDED, SSCI, A&HCI, ESCI Timespan=All years* |
|  |  |
| # 24 | TS=(technolog*) |
|  | *Indexes=SCI-EXPANDED, SSCI, A&HCI, ESCI Timespan=All years* |
|  |  |
| # 23 | TS=((computer or multimedia or digital* or online) NEAR/4 (program* or education* or environment* or learn* or interface* or instruction* or based or platform* or device* or app or apps or application*) ) |
|  | *Indexes=SCI-EXPANDED, SSCI, A&HCI, ESCI Timespan=All years* |
|  |  |
| # 22 | TS=(game* or gaming or gamification or gamify or videogame* or "video game*") |
|  | *Indexes=SCI-EXPANDED, SSCI, A&HCI, ESCI Timespan=All years* |
|  |  |
| # 21 | TS=(high-tech*) |
|  | *Indexes=SCI-EXPANDED, SSCI, A&HCI, ESCI Timespan=All years* |
|  |  |
| # 20 | TS=(telemedicin* or tele-medicin* or telehealth* or tele-health* or telecare* or tele-care* or telecari* or tele-cari* or emedic* or e-medic* or ehealth* or e-health* or mhealth* or m-health* or ehomecare* or e-homecare* or e-home-care* or teleconsultation* or tele-consultation* or videoconsultation* or video-consultation* or telenurs* or tele-nurs* or teletherap* or tele-therap* or telerehab* or tele-rehab* or erehab* or e-rehab* or telemonitor* or tele-monitor*) |
|  | *Indexes=SCI-EXPANDED, SSCI, A&HCI, ESCI Timespan=All years* |
|  |  |
| # 19 | TS=(phone or phones or telephon* or smartphone* or smart-phone* or cellphone* or cell-phone* or mobilephone* or mobile-phone* or "personal digital assistant*" or palmpilot* or palm-pilot* or smarthome* or smart-home* or tablet or tablets) |
|  | *Indexes=SCI-EXPANDED, SSCI, A&HCI, ESCI Timespan=All years* |
|  |  |
| # 18 | TS=(telecommunicat* or tele-communicat* or teleconferenc* or tele-conferenc*or app or apps or app-based or mobile-based or "Short Message Service*" or sms or textmessag* or text-messag* or texting or videoconferenc* or video-conferenc* or webconferenc* or web-conferenc* or webcast* or web-cast* or webinar* or web-application* or web-based-application*) |
|  | *Indexes=SCI-EXPANDED, SSCI, A&HCI, ESCI Timespan=All years* |
|  |  |
| # 17 | TS=(((wearable or wireless) NEAR/1 (technolog* or electronic) ) or (digital NEAR/1 medicine) or (technolog* NEAR/1 health) or ((mobile or internet or electronic* or robot*) NEAR/1 (consultation* or application or applications) )) |
|  | *Indexes=SCI-EXPANDED, SSCI, A&HCI, ESCI Timespan=All years* |
|  |  |
| # 16 | #15 OR #14 OR #13 |
|  | *Indexes=SCI-EXPANDED, SSCI, A&HCI, ESCI Timespan=All years* |
|  |  |
| # 15 | TS=("e-simulation*" or esimulation* or VR) |
|  | *Indexes=SCI-EXPANDED, SSCI, A&HCI, ESCI Timespan=All years* |
|  |  |
| # 14 | TS=((web or online or computer*) NEAR/2 simulation*) |
|  | *Indexes=SCI-EXPANDED, SSCI, A&HCI, ESCI Timespan=All years* |
|  |  |
| # 13 | TS=((virtual* or augmented) NEAR/1 (realit* or environment or environments or room* or patient* or simulation*) ) |
|  | *Indexes=SCI-EXPANDED, SSCI, A&HCI, ESCI Timespan=All years* |
|  |  |
| # 12 | #11 OR #10 OR #9 OR #8 OR #7 OR #6 OR #5 OR #4 OR #3 OR #2 OR #1 |
|  | *Indexes=SCI-EXPANDED, SSCI, A&HCI, ESCI Timespan=All years* |
|  |  |
| # 11 | TS=((Practical or practice) NEAR/0 (Lab or labs or laborator*) ) |
|  | *Indexes=SCI-EXPANDED, SSCI, A&HCI, ESCI Timespan=All years* |
|  |  |
| # 10 | TS=(Learning NEAR/1 (Lab or labs or laborator*) ) |
|  | *Indexes=SCI-EXPANDED, SSCI, A&HCI, ESCI Timespan=All years* |
|  |  |
| # 9 | TS=(skill* NEAR/1 (Lab or labs or laborator*) ) |
|  | *Indexes=SCI-EXPANDED, SSCI, A&HCI, ESCI Timespan=All years* |
|  |  |
| # 8 | TS=(Anatomic* NEAR/1 Model*) |
|  | *Indexes=SCI-EXPANDED, SSCI, A&HCI, ESCI Timespan=All years* |
|  |  |
| # 7 | TS=(skill* NEAR/1 training*) |
|  | *Indexes=SCI-EXPANDED, SSCI, A&HCI, ESCI Timespan=All years* |
|  |  |
| # 6 | TS=("high-fidelity" or "low-fidelity") |
|  | *Indexes=SCI-EXPANDED, SSCI, A&HCI, ESCI Timespan=All years* |
|  |  |
| # 5 | TS=(Standardised NEAR/2 Patient*) |
|  | *Indexes=SCI-EXPANDED, SSCI, A&HCI, ESCI Timespan=All years* |
|  |  |
| # 4 | TS=(Standardized NEAR/2 Patient*) |
|  | *Indexes=SCI-EXPANDED, SSCI, A&HCI, ESCI Timespan=All years* |
|  |  |
| # 3 | TS=(manikin* or mannequin*) |
|  | *Indexes=SCI-EXPANDED, SSCI, A&HCI, ESCI Timespan=All years* |
|  |  |
| # 2 | TS=("Role* play*" OR roleplay*) |
|  | *Indexes=SCI-EXPANDED, SSCI, A&HCI, ESCI Timespan=All years* |
|  |  |
| # 1 | TS=(simulat* OR SBL) |
|  | *Indexes=SCI-EXPANDED, SSCI, A&HCI, ESCI Timespan=All years* |
