# Supplementary material for: Simulation-Based Learning Supported by Technology to Enhance Critical Thinking in Nursing Students: Scoping Review
Source: J Med Internet Res. 2025 Feb 18;27:e58744. doi: 10.2196/58744 (PMC11888118; doi:10.2196/58744)
Supplement: Multimedia Appendix 3 [file jmir_v27i1e58744_app3.docx]

**Multimedia Appendix 3.** Characteristic of the included studies.

| **Author, year, country** | **Aim** | **Study population and sample size** | **Theoretical framework stimulation of CT** | **Technological solution** | **Design/ Outcome measures related to CT** | **Results** |
| --- | --- | --- | --- | --- | --- | --- |
| Albagawi et al. [131] (2024)  Saudi Arabia | To explore the lived experiences of Saudi nursing students in digital clinical experiences | NS (n=21), Sex: 47.6% female, Age range 20-21 | Husserlian Phenomenology | Shadow Health® Digital Clinical Experience | Qualitative phenomenological study/ Content analysis of In-depth interviews | Enhanced critical thinking, comfort, knowledge appraisal, and transition to practice |
| Alharbi et al. [132] (2024)  Saudi Arabia | To assess the relationship between virtual simulation, critical thinking, and self-directed learning abilities | NS in 3^rd^ and 4^th^ year (n=201), Sex: 100%female, Age: Range 20-21 | NR | \| Virtual Simulation \| \| --- \| | Descriptive correlational, non-experimental study/ Critical Thinking Disposition tool | Positive correlation between virtual simulation, critical thinking, and self-directed learning abilities |
| Aller et al. [85] (2023)  USA | To measure nursing student development in decision-making and self-efficacy through computer-based simulations | Junior and senior undergraduate NS (n=50)  Sex: NR  Age: NR | Aller’s Development of Decision-Making and Self-Efficacy Model (ADD-SEM) | Computer-Based Simulations with virtual patients | A multisite, multimethod, cross-sectional design/ Lasater’s Clinical Judgment Rubric (LCJR) | Improved decision-making and psychological capital, with higher scores in noticing and interpreting |
| Badowski et al. [76] (2021) USA | To examine students' perceptions of virtual simulation in meeting their learning needs when compared to traditional clinical experiences and manikin-based simulation environments | NS (n=97)  IG^2^ A (n=47)  IG B (n=28)  IG C (n=22)  Sex: NR^3^  Age: Range 18-48years | Jeffries Simulation Theory | Virtual simulation | A retrospective multi-site exploratory, descriptive study/ The Clinical Learning Environments Comparison Survey (CLECS 2.0) | Virtual simulation met perceived learning needs for CLECS 2.0 subscale items of nursing process, critical thinking, self-efficacy, and teaching-learning dyad |
| Beebe et al. [86] (2024)  USA | To investigate the effect of screen-based simulation on diagnostic reasoning factors (metacognitive awareness, knowledge, and diagnostic reasoning) in Family Nurse Practitioner students during their final semester of clinical coursework | Master’s degree  NS (n=72)  Sex: 90.3% female  Age: mean 34.5 years | Metacognitive Diagnostic Reasoning (MDR) Model© | Screen-Based Simulation using the iHuman Patients© platform | Quasi-experimental, pretest-posttest one group design/ Metacognitive Awareness Inventory, Diagnostic Readiness Test and diagnostic reasoning through mean iHuman case play scores | Screen-based simulation significantly enhanced metacognitive awareness, knowledge, and diagnostic reasoning in Family Nurse Practitioner students, with large effect sizes and meaningful improvements across all measures |
| Blakeslee [51] (2020)  USA | Evaluate high-fidelity simulation's impact on nursing students' CT^4^ skills by examing whether there were statistically significant differences existed in mean CT skill scores within groups and between groups | NS (n=69)  SG^5^ (n=36)  WCSG ^6^ (n=33).  Sex: 84% female  Age: 18-54 years | Cognitive learning theory framework by Aubel and Paul-Elder CT model | High-fidelity simulator Laerdal SimMan 3G© | A quantitative causal-comparative methodology with a pretest post-test design/ Health Science Reasoning Test (HSRT) | No statistically significant difference in participants' mean CT scores within groups or between groups |
| Blanié et al. [136] (2020)  France | To compare the respective educational value of simulation by gaming, and a traditional teaching method to improve CR^7^ skills necessary to detect patient deterioration | NS in 2^nd^. year (n=146)  IG (n=73)  CG^8^ (n=73  Sex: 81% female  Age: Mean 24 (±6.4) years | Theoretical learning model of CR, the script theory | Serious game (LabForGames Warning) | RCT^9^ /Script Concordance Test (SCT) | Although all NS in CG experienced increased CR, no statistically significant difference was found between the IG and CG regarding CR skills scores. NS in IG expressed more satisfaction toward the training session than those in the CG |
| Brown & Chronister [56] (2009) USA | To examine the effect of simulation activities on CT and self-confidence in an electrocardiogram nursing course | NS: (n=140)  Sex: 89% female  Age: Mean 27.5 years  IG (n=70)  CG^8^ (n=70 | Benners Theory | Computer-based simulation; High fidelity simulation manikin as patient (Laerdal’s SimMan) | Correlational research design/critical thinking measured with customized multiple-choice questionnaire | CT measures showed no significant differences between the groups, except when controlled by semester level. The sample variables ´second-semester senior´ and ´job related telemetry experience´ showed significantly effect on CT scores |
| Burns et al. [70] (2010)  USA | To Test high-fidelity simulation for teaching problem-solving skills to 1^st^ year NS students | Undergraduate NS (n=125)  Sex: 92% female  Age: 18-22 years | Nursing process model | High fidelity simulation manikin as patient (Laerdal’s SimMan) | Prospective pre- and post- test design/problem-solving skills | 114 students demonstrated an improvement in CT skills for use in patient care |
| Calik & Kapucu [121] (2024) Turkey | To compare the effectiveness of a serious game and standardized patient simulation in improving nursing students’ knowledge, critical thinking, problem-solving skills, satisfaction, and self-confidence. -solving skills | NS in 2 ^nd^. year (n=72)  Sex: 88.8% female  Age: NR | Gagne’s nine events of instruction and Bloom’s taxonomy | Serious Game (MetaHost) | Quasi-experimental study and 4-week follow-up/ Critical Thinking Disposition Scale; Problem-Solving Inventory | Significant improvements in knowledge, satisfaction, and self-confidence for both groups. Serious Game group showed higher knowledge posttest scores, but no significant differences in critical thinking or problem-solving between groups |
| Chircop et al. [123] (2022)  Canada | To evaluate how Sentinel City virtual simulation for community health nursing, contributes to the learning and development of NS in different locations. To measure its impact on enhancing students' knowledge, skills, and CT abilities in community health nursing | NS: (n=191)  Sex: Most identified as female  Age: Range 21-25 | Constructivist and experiential learning concept | Virtual simulation program; Sentinel City | Mixed methods/ Self developed questionnaire to measure knowledge and CT | The students demonstrated high confidence in their ability to improve their knowledge and  critical thinking after the use of SC. Almost all students (93.62%) were confident and “very confident” in their ability to recognize health inequities, indicating the highest level of confidence (mean = 4.38, ± 0.71) |
| Cieslowski & Haas [77] (2023)  USA | To enrich clinical learning by developing an innovative strategy to deploy immersive VR^10^ in a senior-level advanced laboratory technologies course in a traditional baccalaureate prelicensure program | NS (n=110)  Sex: 89% females  Age: NR | Jefferies Simulation Theory | Immersive VR | Experimental study/ qualitative analysis of students written reflections | Many students used the term critical, emphasizing critical thinking when reflecting on the patient with chronic obstructive pulmonary disease |
| Cole [87] (2024)  USA | To evaluate the impact of a High-fidelity Patient Simulation series on learning, confidence, and clinical reasoning in pediatric nursing education | Undergraduate NS (n=124)  Sex: 83.06 % female  Age: Range 21-28 years | Quality and Safety Education for Nurses (QSEN) framework and core competencies | HPS (High-fidelity Patient Simulation) | Mixed Methods/ Simulation Effectiveness Tool-Modified (SET-M) for confidence and learning | NS rated High-fidelity Patient Simulations significantly higher than clinical experiences in 10 of 12 categories, with notable improvements in confidence, clinical reasoning, and decision-making (Cohen's d = 0.68–0.79). Simulations provided a safe environment for practicing complex scenarios, enhancing learning consistency compared to the variability of clinical experiences |
| Donovan et al. [53] (2018)  USA | To examine undergraduate NS perceptions and experiences when given a computer-based simulation program as a preparation prior to their simulated lab experience | Senior undergraduate NS (n=82)  Sex: 94% female  Age: 20-25 years | Adult learning theory. | Computer-based simulation; vSim | Mixed methods/ Self developed surveys and thematic analysis of focus group interviews | Quantitative and qualitative data showed that the simulator stimulated the students to engage and apply and CT skills. |
| Dubovi, I. [140] (2019)  Israel. | To compare the effectiveness of online computer-based simulations designed using three alternative multimedia approaches (video, animation, and agent-based visualizations) on learning of CR skills | Undergraduate NS (n=97)  Sex: 79.3% female  Age: Mean 22.4 (±1.9) years. | The theory of cognitive engagement called ICAP (Interactive, Constructive, Active, and Passive) | Simulation with video and animation. | Prospective pre- and post-test, time-series design/ Clinical Reasoning Evaluation Tool | Total post-test scores across all topics were significantly higher than the pretest scores. These findings clearly support that online simulations promote CR |
| Durmaz et al. [118] (2012)  Turkey | To examine the effects of screen-based computer simulation (SBCS) on knowledge, skill, and CD-M^11^ in teaching preoperative and postoperative care management | NS in 2^nd^. year (n=82)  Sex: 90.2% female  Age: Mean 20.5 (± 1.1) years.  IG: (n=41)  CG: (n=41) | Information processing theory. | Screen-based computer simulation (SBCS). | RCT/Clinical Decision Making in Nursing Scale | CD-M perceptions of the students were at the medium level, and they were proved to have been developing gradually. No significant differences in knowledge or CD-M; skill scores improved significantly in experimental group |
| Edeer & Sarikaya [120] (2018)  Turkey | Explore NSs’ perceptions of screen-based simulation for skill training.. | NS in 2^nd^. year (n=24)  Sex: 83.3% female  Age: Mean 20.2 (±0.8) years | Information-processing theory | Screen-based computer simulation (SBCS) | A qualitative study, phenomenological approach/ Content analysis of focus group interviews | Most students stated that the SBCS on clinical practice made their decision-making more effective |
| Elcokany, N. M. et al. [130] (2021) Saudi Arabia | To investigate and evaluate the impact of computer-based scenarios on undergraduate NS’ decision-making skills | NS (n=112)  IG: (n=78)  CG: (n=34)  Sex: 100% female  Age: Range 20-23 | Technology Acceptance Model (TAM) Constructivist learning theory/paradigm. | Computer software | A quasi-experimental non-equivalent CG research design/ Decision Making Skills Worksheet | The IG scored significantly higher in their decision-making skills when compared to the CG. In addition, the IG reported that they highly agreed that their general learning and specific nursing abilities improved after using computer-based case scenarios |
| Elliott & Firkins [88] (2024)  USA | To evaluate the use of a scenario-based gamification activity and its impact on student nurses in an accelerated bachelor program’s critical thinking and confidence in acute situations | Undergraduate NS (n=10)  Sex: NR  Age: NR | Gamified learning and adult learning theories | Choose Your Own Pathway (CYOP) activities | Qualitative study using forums/ Content analyses of focus group interviews | The CYOP’s engaged students thinking and led to use of clinical judgement through realism and prioritization of nursing decisions, (2) the CYOP’s interactive engagement facilitated knowledge application through adaptive learning not memorization, and (3) the CYOPs technology design interfered with learning |
| Everett-Thomas et al. [61] (2021)  USA | To assess student nurses’ clinical documentation and CT skills using virtual patients and a simulated electronic health record system | 1^st^ semester undergraduate NS (n=84)  Sex: 95% female  Age: Mean 26.7 (±3.2) years | Benner’s Theory | Virtual simulation and electronic health records (EHR) | Quantitative longitudinal/ Document analysis | There were significant gains in clinical documentation and CT skills between the first and second assignment |
| Fawaz et al. [141] (2016)  Lebanon | To examine the impact of using high-fidelity simulation on the development of CJ^12^ and motivation among Lebanese NS | NS (n=56)  Sex: 58.9 %female (n=33)  Age: Mean 19.1 years. | Tanner’s Clinical Judgment Model | High-fidelity simulation (SimMan) | Post-test, quasi-experimental study design/ Lasater Clinical Judgment Rubric Questionnaire | NS exhibited significant improvement in CJ due to exposure to high-fidelity simulation. There was a significant difference post high-fidelity simulation between the IG and the CG in CJ. IG had a higher mean score of CJ than the CG |
| Fero et al. [54] (2010)  USA | To examine the relationship between  metrics of CT skills and performance in simulated clinical scenarios. | NS (n=36)  Sex: 83.3% female  Age: 20-54 years (63.9% 20-30 years) | Adaptation of Argyris’ and Schon’s theories of Action Espoused, what people say they will do, and Theory-in-Use, what people actually do | Videotaped vignettes, high-fidelity human simulation, a Laerdal SimMan® | Quasi-experimental, cross-over design/ California Critical Thinking Disposition Inventory and California Critical Thinking Skills Test | The relationship between videotaped vignette performance and CT disposition or skills scores was not statistically significant, except for problem recognition and overall CT skills scores. There was a statistically significant relationship between overall high-fidelity human simulation performance and overall CT thinking disposition scores |
| Finn & Bradley [109] (2023) Australia | To explore and evaluate student perceptions and experiences of virtual simulation using vSimÒ for Nursing Gerontology and its impact on learning and preparation for professional experience placement in Primary Health | NS (n=169)  Sex: NR  Age: Range 18-35 | Inquiry-based learning combined with virtual simulation | vSim® for Gerontology | Descriptive convergent mixed methods/ Adapted vSim user feedback survey with additional dditional open-ended questions | vSim® Gerontology program effectively enhanced nursing students' CR and preparation for practice, particularly during COVID-19 disruptions. Overall, vSim® was a valuable supplement to traditional learning methods, supporting readiness for professional experience placements |
| Fogg et al. [89] (2023)  USA | To determine the impact of commercially designed screen-based simulations on students’ performance across C-DM elements of the National Council for State Boards of Nursing (NCSBN) Clinical Judgment Measurement Model (CJMM) | Junior NS (n=68)  Sex: NR  Age: NR | NCSBN Clinical Judgment Measurement Model (CJMM) | Screen-based simulation | Quasi-experimental posttest design/ NCSBN Clinical Judgment Rubric. | Screen-based simulations improved nursing students' ability to select and prioritize interventions but revealed challenges in triaging and patient prioritization, highlighting the need for targeted practice. While SBS effectively enhanced clinical decision-making skills |
| Fogg et al. [67] (2020)  USA | To evaluate the CJ skills of undergraduate baccalaureate NS enrolled in a pediatric nursing course using virtual simulation | Undergraduate NS (n=234)  Sex: NR  Age: NR | NR | Pediatric virtual simulation program (vSim for Nursing). | A repeated measures pilot study design/ Lasater Clinical Judgment Rubric for self-assessed clinical judgment | Results show statistically significant findings in attempts and in student self-perception of their CJ abilities |
| Frost et al. [106]  (2020)  Australia | To explore the contemporary application, inclusive of advantages and challenges, of mixed reality (MR) technology in the education of NS and, its contribution to enhance learning | NS in 3^rd^. year (n=96)  Sex: NR  Age: NR | NR | Microsoft Hololens | Descriptive evaluation design/ Self-developed questionnaire with open-ended questions. Content analysis of qualitative data | The key themes of engagement in learning, and developing CJ emerged from students’ responses, and demonstrated ways in which students felt MR enhanced their learning |
| Fung et al. [137] (2021) Hong Kong | To evaluate the effect of a virtual simulation education programme with debriefing in undergraduate NS | Final year undergraduate NS(n=188)  Sex: 71.3 % female  Age: NR | The debriefing process was developed from the simulation-teaching framework from Kolb's Experiential Learning Theory | Virtual simulation | One-group pre- and post-test design/ CLECS | CT were applied better in the traditional clinical environment than the simulated environment, this was significant, p<.05. (N=181 and N=157 in the CLECS critical thinking subscale t-test analysis) |
| Goodstone et al. [64] (2013)  USA | To explore the development of CT for students who received instruction using high-fidelity patient simulation (HFPS) versus low-fidelity simulation (instructor-written case studies) | NS (n=42)  Sex: 73.8% female  Age: mean 27 (±10) years  SG: (n=20)  WCSG: (n=22) | NR | High-fidelity simulation | A two-group quasi-experimental study. pre- and post-test/ Health Studies Reasoning Test | Both groups showed an increase in CT skills. There was no statistically significant difference between the HPS and case study groups |
| Hamidi et al. [124] (2024)  Canada | To explore the perceived effectiveness of a Suicidal Ideation Assessment of Risk virtual simulation module for undergraduate nursing students | NS in 3^rd^. year (n=130)  Sex: 93.1% female  Age: Range 18-31 years | NLN/Jeffries Simulation Theory | Virtual simulation module | Mixed-methods explanatory sequential design/ Simulation Effectiveness Tool-Modified  (SET-M) | Quantitative findings revealed that the virtual simulation module was effective in increasing students' confidence, preparedness, and clinical decision-making abilities. Qualitative findings identified increased learning, preparedness, confidence, knowledge, critical reflection, and decreased anxiety |
| Han & Jin [112] (2024)  China | To evaluate the combination of virtual and in-person simulations among undergraduate nursing students. | Junior NS (n=93), IG (n=45), 64 % female  CG (n=48), 62.5% female  Age: NR | The NLN Jeffries simulation theory | CBS: vSim combined with in person simulations with HPS | Mixed methods QUAN→qual sequential explanatory approach/ CLECS | Combined simulation improved knowledge and non-technical skills (decision-making, critical thinking) compared to in-person simulation alone. |
| Havola et al. [129] (2021)  Finland | To evaluate the effects of two kinds of simulation games, a computer-based simulation and a VR simulation, on self-evaluated CR skills by NS | Undergraduate NS (n=40)  Sex: NR  Age: NR | The CR Cycle by Levett-Jones et al. (2010) | Computer-based simulation game and a VR simulation. | A one-group pretest and post-test design/ Clinical Reasoning Skills scale (CRSs) | NS' self-evaluated CR skills were systematically improved both after playing the computer-based simulation game and after the VR simulation session even though statistically significant differences between self-evaluated CR skills were found only in a few items. |
| Henrichs et al. [82] (2002)  USA | To describe the perceptions of nurse anesthesia students (NAS) who used a MedSim simulator as part of their educational training | NAS NS (n=12)  Sex: NR  Age: NR | NR | Computer driven manikin. MedSim simulator | Qualitative study/ Content analysis of focus group interviews | The NAS felt that the simulator has several advantages. It helps develop CT and decision-making skills. Students felt the simulator could be used to evaluate their own cognitive and psychomotoric skills |
| Hosseini, T. M. et al. [139] 2022, Iran | To investigate the effects of using a virtual patient simulator on the acquisition of CD-M skills in NSs during the pandemic COVID-19 | NS in 3^rd^. year (n=58)  Sex: 81.7 % females  Age: Mean 21 (± 4.5) years | The National League for Nursing Jeffries Simulation Theory, combined with International Nursing Association for Clinical Simulation and Learning (INACSL) Standards of Best  Practice (SOBP) 22 | Cyberpatient software. | Quasi-experimental study with a post-test design/ CD-M Instrument by Lauri & Salantera | CD-M skills of NSs was compared before (48/04 ± 12/77) and immediately after training (91/49 ± 7/66) using paired tests, and a statistically significant difference was found (P = 0/009). A statistical difference was also observed in CD-M skills before and after one month of follow-up (P = 0/001). Also, before the intervention, most students were thinking analytically (63/80%) and making clinical decisions, while after the intervention, most students had an analytic-intuitive model of CD-M (63/80%) |
| Hudson & Penkalski [74] (2022)  USA | To evaluate the effectiveness of high-fidelity simulation (HFS) and interactive case studies (ICS) as teaching strategies for practical NS. To determine if HFS produced higher CT scores compared to ICS. To determine if other, less costly, pedagogical strategies resulted in similar outcomes. How simulation affects CT | NS (n=29)  Sex: 92.8% female  Age: mean 28 | Benner's theory combined with Kolb’s Experiential Learning Theory | High-fidelity simulation and ICS. | Quasi-experimental two group, pre-post-test study/ The Assessment Technologies Institute (ATI) fundamentals specialty examination | Both interventions had significant results (p = .001) between pretest and post-test; however, significance was not found regarding post-test scores between the HFS and ICS |
| Hwang & Chang [133] (2020)  Taiwan | To explore the situation of intravenous injection flipped learning and the effectiveness of the game in a nursing school in northern Taiwan | NS in 2^nd^.year (n=56)  Sex: NR  Age: Mean 20 years | NR | RPG Maker MV, developed by Enterbrain | Quasi-experimental study design/ The critical thinking tendency scale | The students learning with RPG Maker MV approach showed better intravenous injection comprehensions well as higher learning achievement, learning motivation, learning attitudes, flow experience and CT tendency than those learning with conventional flipped learning |
| Jeong et al. [98] (2022)  South Korea | To establish and evaluate the effectiveness of a VR simulation program using COVID-19. | NS (n=65)  IG: (n=32), 75% female  CG: (n=33), 90.9% female  Age: NR. | NR | VR simulation program. | Quasi-experimental study using a non-equivalent control group pre-post-test design/ Korean CR capacity | The IG exhibited no statistically significant alteration in CR capacity when compared to the CG) (t = 0.27, p = .778). However, statistically significant differences were observed between pretest and post-test measurements |
| Jung & Roh [99] (2022)  Republic of Korea | To determine whether the cognitive load has a mediating effect on the relationship between learning flow and CR skills of NSs in vSim learning | NS (n=148)  Sex: 88,5 % females  Age: Mean 23.4 (±3.38) years | NR | Virtual Simulation (vSim)-Based Learning Experience | Cross-sectional study/ CR skills scale | Students’ learning flow (β= 0.657, SE = 0.078, p < .001) and cognitive load (β= 0.137, SE = 0.054, p = .044) were significant predictors of their clinical reasoning skills |
| Kang et al [93] (2020)  Korea | To investigate the impact of virtual simulation on NS’ CT and self-directed learning abilities | Senior NS (n=47)  Sex: 100% female  Age: Mean 23.74 (±1.32) years | NR | Virtual simulation program vSim for Nursing | Quasi-experimental one-group pretest - post-test/ Critical-Thinking Disposition scale | No statistically significant differences emerged between the assessments on CT or self-directed learning ability before and after virtual simulation |
| Karaduman & Basak [119] (2023) Turkey | To compare the effects of virtual and human patient simulation methods on performance, simulation-based learn- ing, anxiety, and self-confidence with clinical decision-making scores of nursing students | NS (n=126)  IG 1: (n=42), 66.7 % female, mean age 21 years  IG 2: (n=42), 69% female, mean age 20.76 years  IG 3: (n=42), 78.6% female, mean age 21,33 years | Kolb´s Experiential Learning Theory | Virtual simulation | Quasi-experimental, stratified, RCT/ Nursing Anxiety and Self-Confidence with CD-M Scale | Performance scores were statistically significant higher in the virtual patient simulation group (P < .001). It was determined that virtual patient simulation was superior to other methods in terms of nursing anxiety and self-confidence with clinical decision- making, simulation-based learning, and performance scores |
| Kiegaldie & Shaw [110] (2023)  Australia | To evaluate the effectiveness and feasibility of using a VRS program, JasperVR, to enhance the learning outcomes, motivation, and self-efficacy of nursing students compared to traditional simulation methods | NS (n=675)  Sex: 77.5% female  Age: 18–25 years (65.6%)  26–50+ years (32,1%)  IG (n=393),  CG (n=282) | NR | Immersive virtual simulation | Mixed-methods quasi-experimental design/ Content analyses of focus group interview or Individual telephone interviews | JasperVR, a VR simulation program, significantly enhanced NSs' learning outcomes, including CR, CD-M, and confidence in clinical situations. The findings support that VR simulation can be a valuable approach to preparing nursing students for clinical challenges and bridging the gap between theory and practice |
| Kim et al. [101] (2024)  South Korea | To investigate the impact of VR-based mental health nursing simulation on student competence | NS (n=50  Sex: NR  Age: NR) | NR | VR-Based Mental Health Nursing Simulation | Pre-post experimental design/ Problem-Solving Process; Critical Thinking Ability | Significant improvements in critical thinking, problem-solving, communication, and attitudes toward mental health care |
| Kim & Kim [94] (2015)  Korea | To assess the effects of the addition of a one-time simulation experience to the didactic curriculum on NS knowledge acquisition, CR skill, and self-confidence | Junior NS (n=94)  IG: (n=48), 0.87% female, mean age 21.06 (±1.80) years  CG: (n=46), 89.1 % female, mean age 20.98 (±1.35) years | NR | High-fidelity SimMan® | A quasi-experimental crossover (pretest-post-test) design/ nursing process model-based rubric | Students IG scored significantly higher on CR skill and related knowledge than those in CC |
| Kjernan & Olsen [72] (2020)  USA | To discuss and assess the perceived competency of junior and senior NS in clinical behaviors and skills | NS (n=62)  First-semester juniors: (n=27), 85.1% female, mean age 23.9 years  First-semester seniors: (n=35), 80% female, mean age 26 years | NR | Manikin based simulations and HPSs | Quasi- experimental design with pre- and post-test/ clinical  competency questionnaire (CCQ) | Both groups saw a large improvement in taking precautions to minimize risks to patients. The largest improvement for juniors addressed CT and accepting constructive criticism |
| Kleinheksel [83] (2014)  USA | To assess the quality of the reflections of Master of Science (Msc) student written with in the Digital Clinical Experience (DCE) and explore which variables may influence successful critical levels of reflection | Msc NS (n=130)  Sex: NR  Age: NR | Situated cognition and transformative learning theory. Under the constructivist paradigm | Shadow Health DCE | Exploratory study within-stage mixed-model design/ Data NS in the DCE software was analysed | Critical self-reflection indicating transformative learning was predicted by the number of secondary clinical items students uncover during their virtual patient interview. This level of self-reflection did not occur for. students who discovered only the primary clinical findings |
| Koivisto et al. [127] (2016)  Finland | To investigate NS experiences of learning CR process by playing a 3D simulation game | NS (n=166) from the surgical nursing course  Sex: NR  Age: 15-55 years | Kolb's Experiential Learning Theory | 3D simulation computer game | Cross-sectional descriptive study/ Self-developed questionnaire | Application of nursing knowledge and learning of the CR process showed a moderate or strong positive correlation |
| Koivisto et al. [128] (2016)  Finland | To describe and explain how NS can learn CR by playing a simulation game | NS (n=166)  Sex: NR  Age: Range 21-25 years | The CR Cycle by Levett-Jones et al. (2010) | The prototype of the CareMe simulation game | Pre-post-test study design/ Self-developed questionnaire | Usability application of nursing knowledge, and exploration have the most impact on learning CR when playing simulation games. Findings also revealed that authentic patient-related experiences, feedback, and reflection have an indirect effect on learning clinical reasoning |
| Kuiper et al. [84] (2008)  USA | To explore the impact of patient simulation technology on situated cognition of undergraduate NS with the long-term goal of preparing a workforce of practitioners who effectively manage clinical issues | Undergraduate senior NS (n= 44)  Sex: 89% female  Age: Mean 22 years | Situated cognition, social cognitive theory with constructivism as the underlying philosophical approach | High-fidelity patient simulation | Descriptive design/ Qualitative analysis from NS’ narrative experiences | NS related that their experience of think independently and challenged their CD-M skills. There was no significant difference between authentic clinical experiences and high-fidelity patient simulation |
| Lasater [81] (2007) USA | To examine the experience dimension, that is, the high-fidelity simulation experiences of some of the first student participants and the effect of the experiences on the students’ development of CJ | Junior-level NS (n=48)  Sex: 85% female  Age: 24-50 years. | The Lasater Interactive Model of CJ | Computerized human patient simulator | Qualitative retrospective study design/ Content analysis of focus group interview | Simulation was frequently praised by for integrating theoretical knowledge, psychomotor skills, and clinical practice, CT. Another group noted that, although scenarios were extreme, they appreciated how these encouraged anticipating clinical events, particularly during medication administration, enhancing CJ |
| Lee & Baek [102] (2023)  South Korea | To develop and evaluate the effectiveness of a VR simulation nursing education program related to postoperative patient nursing based on an information processing model | NS in 3^rd^. year (n=44)  IG (n=22), mean age 21.13 (± 1.01) years  CG (n=22), mean age 21.30 (± 0.87) years.  Sex: NR | Information Processing Model | Web-based VR and High-fidelity simulation | Quasi-experimental, pretest-posttest design with control group/ Clinical Decision-Making in Nursing Scale (CD-MNS) | The information processing model–based VR simulation nursing education program was effective in improving nursing students' performance confidence and clinical decision-making ability |
| Lee & Baek [104] (2024)  South Korea | To develop and evaluate VR-based adult nursing education programs | NS in 4th year (n=44)  IG (n=22)  CG (n=22)  Sex: NR  Age: NR | The information processing model | CBS: Web based VR: VS nursing ver. 2.0 program on laptop, tablet or smartphone-celle | Non-quantitative control group pretest–post-test design/ Problem-Solving Ability; Critical thinking disposition scale for nursing students | Enhanced critical thinking, problem-solving, and confidence in nursing tasks |
| Lee & Han [95] (2022)  Sør-Korea | To develop a mechanical ventilation nursing program using VR and evaluate the effect of knowledge, self-efficacy, CR capacity, learning immersion, and learning satisfaction among NS | NS in 4^th^. year (n=60). CG: (n=30), 90% female, mean age 22.8. (± 1.5) years. IG: (n=30), 96.6% female, mean age 22.2 (± 0.85) years | NR | VR simulation | Quasi‑experiment design/ CR capacity scale | The interaction effect of the intervention time and CG, to which the VR simulation pro‐ gram was applied, showed a significant difference CR capacity (F = 16.97, p < .001) |
| Lee et al. [103] (2023)  South Korea | To develop and evaluate the effectiveness of a VR-based patient severity classification competency promotion program in nursing students during the COVID-19 pandemic | NS in 4^th^. year (n=34)  IG (n=17), mean age 21.65 (± 0.61) years  CG (n=17), ), mean age 21.59 (± 0.87) years  Sex: NR | Analysis-Design-Development-Implementation-Evaluation (ADDIE) model | Virtual simulation and Immersive virtual simulation | Quasi-experimental, pretest-posttest design with control group/ Clinical Decision-Making in Nursing Scale (CD-MNS) | The VR-based nursing education program effectively improved students’ severity classification competency, performance confidence, and clinical decision-making ability. The VR-based nursing education program provides realistic indirect experiences to nursing students in situations where clinical nursing practice is not possible |
| Legge et al. [90] (2024)  USA | To describe an educational innovation employing a virtual clinical simulation using the tenets of Tanner’s Clinical Judgment Model and a National League for Nursing Advancing Care Excellence for Seniors unfolding case | Undergraduate NS (n=86)  Sex: NR  Age: NR | Tanner’s Clinical Judgment Model (CJM) | Virtual simulation and Immersive virtual simulation | Descriptive evaluation study/ self-developed questionnaire delivered by The Qualtrics survey | A virtual clinical simulation successfully served to  replace direct care clinical for 86 undergraduate nursing students enrolled in a foundation nursing course, augmenting  the students’ ability to transfer and apply theoretical knowledge to clinical practice. This virtual clinical simulation served as a viable platform for teaching clinical reasoning and clinical judgment, mitigating the education-practice gap for new graduate registered nurses |
| Lewis & Ciak [71] (2011)  USA | To investigate the impact simulation laboratory experiences have on CT, student satisfaction, self-confidence, and cognitive learning | NS (n=63)  Sex: Female (n=63)  Age: Mean 28 years | NR | High-fidelity simulators | Quasi- experimental design/ Nursing Care of Children and Maternal Newborn test | No definite results regarding CT. On some CT components (e.g., evaluation and explanation in maternal-newborn and analysis in care of children), scores were higher for the IG than for the CG. The opposite was true for other CT areas (e.g., inference and analysis in maternal-newborn and evaluation and explanation in nursing care of children) |
| Li et al. [114] (2023)  China | To verify the teaching effectiveness of VR-based cognitive assessment and  rehabilitation simulation courses by the VSCERTS (Virtual Supermarket Cognitive Evaluation and Rehabilitation Teaching System) and the effects on the critical thinking ability of undergraduate nursing students | NS in 3^rd^. year (n=84)  Sex: 65% female  Age: 18 years and above | Authentic Learning | Virtual simulation and Immersive virtual simulation | Quasi-experimental, pretest-posttest design/ Critical Thinking Disposition Inventory (CTDI-CV) | The VR-based cognitive assessment and rehabilitation simulation course using the VSCERTS can significantly improve the critical thinking ability of nursing undergraduates and provide a novel and effective teaching method for nursing education |
| Li et al. [115] (2024)  China | To explore the impact on NSs’cardiopulmonary  Resuscitation CPR skills after participation in online virtual simulation with interactive exercises and offline low-  fidelity simulation | NS in 1^st^. year (n=72)  IG (n=36), 80.6% female, mean age 18.69 (± 1.91) years  CG (n=36), 77.8% female, mean age 18.25 ( ± 0.55) years | Constructivist theory | Virtual simulation and Immersive virtual simulation | RCT/ Chinese Critical Thinking Dispositions Inventory (CCTDI-CV) | The IG demonstrated significantly greater improvement in their self-directed learning (SDL) abilities and CPR skills, whereas no statistically significant differences were observed in overall CT abilities between the IG and CG. |
| Lowdermilk & Fishel [59, 111] (1991)  USA | To evaluate the use of Computer-assisted instruction (CAI) clinical simulations as a strategy for teaching, enhancing, and evaluating NS' CD-M skills | NS (n=64)  Sex: NR  Age: NR | NR | CBS -CAI | RCT with pre and post-test/ Kolb's Learning Style Inventory (LSI) and Score evaluation of Computer-Assisted Instruction (CAI) | There was no difference between the students completing the full CAI series and those who did not. Students who made significant improvements in decision-making scores also earned higher clinical grades |
| Luo et al. [104] (2021) China | To understand students’ performance, learning effectiveness, and satisfaction with their participation in distance learning. To compare the differences in students’ performance, learning effectiveness, and satisfaction between male and female students | NS in 4^th^. year (n=35)  Sex: 71.4% females  Age: Mean 21.80 (±1.2) years | NR | Virtual simulation with webinars (online) | Descriptive and quasi-experimental design/ Clinical Thinking Ability Scale | A statistically signiﬁcant improvement in the students’ clinical thinking ability was observed after performing the virtual simulation (p = .001). After the virtual simulation, the female students showed better abilities in all the dimensions of clinical thinking ability |
| Maheu-Cadotte et al. [125] (2023)  Canada | To evaluate to serious game’ prototypes contribution to clinical reasoning in acute heart failure cases | NS (n=28)  Sex: 93% female  Age: median 24 years | Game-Based Learning Model | Serious Games (SIGN@L) | Multimethod design/ Clinical reasoning using self-reported questionnaires and Content analysis of semi structured interview | Higher engagement and intrinsic motivation was reported; clinical reasoning showed negligible differences across prototypes |
| Mahoney et al. [63] (2013)  USA | To explore how high-fidelity patient simulation in pediatric nursing education with a focus on clinical reasoning (CR) impacts critical thinking (CT) | Baccalaureate NS (n=131)  Fall 2009 (n=64)  Spring 2010 (n=69)  Sex: NR  Age: NR | Benner's theory | High fidelity patient simulators (Laerdal SimMan®), 2 infants (Laerdal SimBaby), and 1 adult simulator with the capability of pregnancy simulation (Vital Annie) | A quasi-experimental post-test design/ Content analysis of qualitative data | Data revealed that learning objectives were met over 80% of the time in simulation exercises and the qualitative themes revealed a positive experience with the simulation exercises including improved critical thinking skills |
| Manik et al. [138] (2022)  Indonesia | To describe Indonesian NS’ perspectives regarding the use of vSim for Nursing^TM^ | NS (n=50)  Sex: 84% female (n=42)  Age: Range 18-22 | NR | Virtual simulation vSim for Nursing^TM^ | Mixed-methods approach/ Content analysis of qualitative data | Learning to think critically was expressed repeatedly in the qualitative datal and systematically, even virtually |
| McCaughey & Traynor [146] (2010)  UK | To evaluate the role of medium to high fidelity simulation in the students' preparation for clinical practice | Undergraduate NS (n=153)  Sex: NR  Age: NR | NR | Medium to high fidelity simulation | Longitudinal Descriptive survey/ self-developed questionnaire | Simulation was judged by 92.5% (n=86) of respondents to increase confidence in their CJ |
| Montenery et al. [80] (2013)  USA | To determine how millennial NS perceive the effects of instructional technology on their attentiveness, knowledge, CT, and satisfaction | Undergraduate NS (n=124)  Sex: NR  Age: NR | Benner's theory combined with Kolb’s Experiential Learning Theory | Audience response, virtual learning, simulation, and computerized testing technologies | Descriptive, longitudinal study/ self-developed questionnaire | Participants reported a positive impact of human patient simulators, indicating that simulators and scenarios enhanced their learning and CT |
| Onay et al. [122] (2024)  Tyrkey | To determine the effectiveness of a virtual service/patient-based program (vSPBP) for nursing education | NS in 4^th^. year (n=95), 56. 8% female  IG (n= 44), 63.6% female  CG (n= 51) 51% Female | Task and Competency-Based Learning | Computer-Based Simulation (CBS) with Virtual Patient-Based Program | Mixed methods, quasi-experimental design/ Clinical Decision-Making in Nursing Scale (CD-MNS | Effective for developing care planning and clinical decision-making skills, with significant improvement in care plan preparation for intervention group |
| Padilha et al. [143] (2019)  Portugal | To evaluate the effect of clinical virtual simulation with regard to knowledge retention, CR, self-efficacy, and satisfaction with the learning experience among NSs. | NS (n=42)  Sex: 95.2% female  Age: Range 19-29.  CG: (n=21)  IG: (n=21) | NR | Clinical virtual simulator: Body Interact | RCT with a pretest and 2 post-tests/ Self- developed true or false and multiple-choice knowledge test | The results indicate that clinical virtual simulation improves 20,4 % of knowledge retention and initial CR over time (2 months) |
| Pardue et al. [75] (2022)  USA | To determine NS’ learning experiences when engaged in VR simulation, and to explore learners’ problem-solving/CR approach after participation in a VR Simulation clinical scenario | NS (n=19)  Sex: NR  Age: NR | Nursing CJ Framework | VR Simulation | Qualitative design/ Content analyses from focus group interview | The NS described that they developed a new awareness, a creation and a self-improvement plan and an opportunity to think about ones thinking and how to improve personal performance. VR Simulation promotes the development of nursing CJ |
| Park et al. [96] (2022)  Korea | To investigate and assess the effectiveness of two different simulation modalities, virtual simulations and high-fidelity simulation s, in enhancing learning and skill acquisition among NS. To determine  differences in 1) the problem-solving process, 2) CR, 3) reflective thinking, 4) satisfaction with the practicum, and 5) self-confidence | NS (n=52)  Sex: NR  IG 1: (n=26), median age 22 years  IG 1: (n=26), median 22 years | NR | Virtual simulation used the vSim® for nursing | Quasi experimental crossover design/ Problem solving process behavior survey; nurses clinical reasoning scale and reflective learning continuum | The virtual simulation-first, HFS-second order led to significantly higher scores for reflective thinking (z = 3.53, p < .001) and self-confidence (z = 2.47, p = .013) than the other order |
| Parr & Sweeney [66] (2006) USA | To share experience with the integration of simulation into a baccalaureate nursing program, student evaluation of the simulation experiences, and suggestions for others planning such a center | Undergraduate NS (n=17)  Sex: NR  Age: NR | NR | 4 adult Human Patient Simulator emergency care simulators (ECS) and a laptop Medical Education Technologies, Inc (METI) | Post-test Survey based evaluation/ Self-developed questionnaire | The item with the highest mean score was the fourth item, stating that the HPS experience challenged thinking and decision-making skills. Using the Wilcoxon signed rank test, the mean score of the fourth item was found to be significantly higher than the mean scores for the other items |
| Peddle et al. [105] (2019)  Australia | To explore undergraduate nursing students' learning of non-technical skills (NTS) following interactions with the virtual patients in the case study | Undergraduate NS (n=71)  NS in 1^st^. year (n=40)  NS in 3^rd^. year (n=31)  Sex: NR  Age: NR | Dignity Model  DT | Virtual patients (VP) | A qualitative design, case study methodology/ Content analysis of focus group interviews | NS reported that interactions with VPs developed knowledge and skills across all categories of NTS including communication, situation awareness, teamwork, decision-making skills and duty, advocacy and empathy to varying degrees |
| Peng & Wu [135] (2024)  Taiwan | To determine the effect of high-fidelity tele-simulation on emergency and and critical patient care-related knowledge, self-confidence, and critical thinking skills in nursing students | NS in 3^rd^. year (n=84)  Sex: NR  IG (n=43), mean age 21.54 (± 0.57) years  CG (n=41)  mean age 21.72 (± 0.70) years | National League of Nursing Jeffries Simulation Theory | High-Fidelity Tele-simulation and Computerized Full-Body Manikin | Randomized controlled trial/ Critical Thinking Intention Scale | Significant improvement in knowledge, self-confidence, and critical thinking for the experimental group |
| Perez et al. [91] (2024)  USA | To compare differences in community health nursing learning outcomes between standardized patient simulation and mannequins | Prelicensure NS (n=66)  IG (n=27)  CG (n=39)  Sex: NR  Age: NR | National League of Nursing Jeffries Simulation Theory | High-Fidelity Manikin Simulation | Cross-sectional study pre-post-test design/ Simulation Evaluation Tool | Standardized patient simulation yielded better clinical reasoning, realism, and satisfaction scores than manikin simulation |
| Powell-Laney et al. [68] (2012)  USA | To investigate if the use of HPS technology leads to increased CD-M ability and clinical performance compared to the teaching modality of a paper and pencil case study | NS (n=133)  Sex: 88% female  Age: Mean 32 years | Dewey’s learning theory | HPS SimMan® | Quasi-experimental/ Scored based on the percentage of correct items regarding patient care | Students in the simulation groups were significantly more likely to score higher on the CD-M exams than students in the case study groups |
| Powers et al. [79] (2020)  USA | To explore baccalaureate NS' experiences with multi-patient, standardized patient simulations that used telehealth to provide opportunities to learn and practice intra- and interprofessional collaboration | NS (n=27)  Sex: 95.3% female  Age: 18-25 years | QSEN Competencies Framework | Simulation and FaceTime technology | Phenomenological qualitative study/ Content analysis of focus group interviews | Activities were identified as helpful for the preparation work and the board games. During the simulations, their CR was enhanced through the facilitator's approach and the assigned roles |
| Ravert [60] (2008)  USA | To assess differences in CT between three groups (simulator (S), non-simulator (N-S), control of students | Undergraduate NS in the first medical-surgical nursing course (n=40)  S (n=12), 100% female, mean age 21,7 years  N-S (n=13), 92.3% female, mean age 22.9 years  CG: (n=15), 100% female, mean age 21.5 years | Kolb’s Experiential Learning Theory | The human Patient simulator marketed by medical education Technologies | Pretest-post-test research design/ california critical Thinking Disposition inventory (CCTDI) and the california critical Thinking skills Test (CCTST) | CT scores of dispositions and skill increased for all three groups, but did not significantly differ between groups |
| Reierson et al. [142] (2015)  Norway | To examine the perspectives of undergraduate NS on telenursing in patient care after simulating three telenursing scenarios using real-time video and audio technology | Undergraduate senior NS (n= 32)  Sex: 93.7% female  Age: Mean 25.4 (±5.2) years | NR | Telenursing simulation in patient care | An exploratory, qualitative desig/ Content analysis of focus group interviews | Telenursing simulation influence nursing assessment and decision making, supporting professional development, particularly by easily allowing a second opinion on nursing assessments |
| Rhodes & Curran [73] (2005)  USA | To describe how faculty used the human patient simulator in creating a case scenario that enhanced CT in senior NS | Senior NS (n=21)  Sex: NR  Age: NR | Benner’s theory | Human Patient Simulator | Experimental study with quantitative approach/ Self-developed questionnaire | Some students experienced it as difficult to treat the HPS as a real patient. Students noted that they were using CT skills |
| Rim & Hyunsook [97] (2022)  Korea | To develop a multi-user virtual simulation program for metacognition and evaluate the students’ satisfaction, CJ, and nursing competencies | NS (n=57)  Sex: 82.2% female  Age: Mean 22,8 (±1.2) years | Experiential learning theory | Multi-User Virtual Environment Simulation (MUVEs) | Mixed methods study/ Lasater Clinical Judgment Rubric questionnaire, Content analysis of focus group interviews | The total scores of CJ in the five scenarios ranged from 22.2 to 32.0 (out of 44 maximum possible points). Participants felt like practicing nurses, employed critical thinking on the patient situation, and practiced priority setting |
| Rush et al. [58] (2008)  USA | To understand the CT of distance RN-to- Bachelor of Science in Nursing (BSN) students who participated in a simulation designed with interactive questions | NS (n=33)  Sex: 100% female Age: Mean 34.3 years | NR | An intermediate-fidelity simulation that combined multimedia technologies and SimMan® | Exploratory qualitative approach/ The data were analyzed using the conceptualization of critical thinking by Scheffer and Rubenfeld | All CT habits of the mind and skills as conceptualized by Scheffer and Rubenfeld (2000) appeared among RN-to-BSN students during the simulation experience |
| Şahin & Başak [117] (2021)  Turkey | To investigate the effect of virtual patient simulation on NSs’ CD-M and problem-solving skills | NS in 4^th^. year (n=73)  Sex: Female (93.2%)  Age: Mean 22.06 (± 0.34) years | NR | Body Interact | Quasi experimental study with a pre-post-test design/ Clinical Decision-Making in Nursing Scale (CDMNS), and the Problem-Solving Inventory (PSI) | NSs have good problem-solving and CD-M skills, and that the administration of virtual patient simulation did not have a direct effect on the improvement of these skills. No significant difference was found between the pre and post-test scores for problem-solving and CD-M skills |
| Schoening et al. [62] (2006)  USA | To examine students’ perceptions of a preterm labor simulated clinical experience as a method of instruction, emphasizing the importance of the educators’ role in promoting positive student outcomes | Baccalaureate NSs (n=60)  Sex: 98.3%, female (n=59)  Age: Mean 22 years | Joyce and Weil’s 4-phase teaching model for simulation | Emergency Care Simulator (Medical Education Technologies, Inc) | Nonexperimental one-group post-test design/ Self-developed questionnaire and content analysis of qualitative data | Simulation allowed the students to have a clinical scenario that was realistic and use their CT skills and knowledge to make decisions as the scenario unfolded |
| Shin et al. [100] (2015)  South Korea | To examine the effect of an integrated pediatric nursing simulation used in a nursing practicum on students' CT abilities and identified the effects of differing numbers of simulation exposures on CT in a multi-site environment | NS (n=237) from pediatric practicum at three universities  Sex: 97% female  Age: Mean 21.9 (±1.6) | Jeffries and Tanner frameworks | High-fidelity simulators | Multi-site, pre-test, post-test design/Yoon's Critical Thinking Disposition tool | The overall CT score significantly increased and NSs with a single exposure to the simulation courseware showed no significant gains in CT, whereas NSs with three exposure showed significant gains in CT |
| Smith et al. [69] (2013)  USA | To determine whether the addition of educational interventions to required clinical hours promotes confidence in triage decision making among NSs enrolled in a final capstone course | Senior-level NS (n=14)  Sex: 78.6% female  Age: 21-49 years | NR | Human patient simulation | Pilot study using an experimental design/ Triage Decision Making Inventory | All groups exhibited higher scores on the Triage Decision Making Inventory from the pretest to the post-test. Students who received both the simulations and the Advanced Cardiac Life Support course demonstrated a significant difference across time |
| Stemer et al. [144] (2023)  Sweden | To explore blended simulation's effect on nursing students' critical thinking skills | NS (n=61)  Sex: 93.4% female  Age: mean 30 (± 7.54) years | NR | High-Fidelity and Web-Based Simulation | Quasi-experimental, one-group pre-post design/CTQ based on California Critical Thinking Disposition Inventory (CCTDI) and Watson-Glaser Critical Thinking Appraisal tool | Significant improvement in critical thinking; large effect size observed between pre- and post-education scores |
| Sullivan-Mann et al. [65] (2009)  USA | To investigate the effect of using simulation as a teaching strategy on the CT abilities of NS | AND NS: (n=53)  Sex: Female (n=50)  Age: mean 25.6 (±5.9) years | Roy Adaptation Model combined with Benner's Novice to Expert model | Human patient simulator Model ECS from Medical Education Technologies, Inc (METI) | Experimental design with a pretest and post-test/ Health Sciences Reasoning Test (HSRT) | Experimental participants showed an increase in CT scores in post-test scores |
| Thiele et al. [78] (1986)  USA | To investigate cue recognition and CD-M abilities of junior and senior baccalaureate NS | NS (n=80)  Sex: NR  Age: NR | Decision theory | Clinical simulations WEIS developed and programmed for use on Apple II microcomputers | Pre-post-test quasi-experimental study design/ Score evaluation of Computer-Assisted Instruction (CAI) | A statistically significant difference was noted in both junior and senior students in relation to accuracy of cue recognition and CD-M |
| Tong et al. [116] China  (2024) | To evaluate the effects of various simulation (high-fidelity simulation (HFS), computer-based simulation, high- fidelity simulation combined with computer-based simulation, and case study) methods on nursing competencies | NS (n=239)  HFS (n=58), 84.4% female, mean age 21, 2 (± 1,7) years  CBS (n=67), 80.5% female, mean age 21.0(± 0.69) years  HFS and CBS (n=57), 80.7% female, mean age 21.1 (± 1.9) years  case study group (n=57), 82.2% female, mean age 20.9 (± 1.4) years | NR | Laerdal patient simulator; Gaumard patient simulators.  High-fidelity simulation (SimMan® 3G).  Computer-based simulation (vSim® for Nursing) | Multicenter randomized controlled trial with pre-posttests and long-term evaluations/ Critical Disposition Scale | HFS and HF+CB simulations were more effective for skill improvement in the short term; all methods equally improved knowledge, collaboration, and critical thinking. |
| Uppor et al. [145] (2024)  Thailand | To investigate the effect  of an Experiential Learning Simulation-Based Learning Program on CJ among nursing  students | Obstetric NS (n=44)  Sex: 86% female  IG (n=22), mean 21. 68 (± 0) years  CG (n=22), mean 2.81 (± 0.66) years | Kolb's Experiential Learning Theory | Experiential Learning Simulation Based Learning Program (ELSBLP) | Quasi-experimental, two-group pre-post-test design/ Clinical Judgment Rubic – Thai version (T-LCJR) Measured CJ using scenario-based questionnaires | Experimental group demonstrated significantly higher CJ scores than the control group |
| Vihos et al. [126] (2024)  Canada | To examine the relationship between VR simulation, satisfaction, and self-confidence in nursing students | NS in 2 ^nd^. year (n=37)  Sex: NR  Age: Range 18-24 | International Nursing Association of Clinical Simulation and Learning (INACSL) best practice standards | Elsevier’s Simulation Learning System with Immersive VR | Mixed methods explanatory, sequential study/ The Student Satisfaction and self-confidence in learning scale (SSLS) Content analysis from semi structured interviews | High satisfaction and self-confidence; themes of fidelity, communication competence, peer learning, critical thinking, and safe learning environment. |
| Volejnikova-Wenger et al. [108] (2021) Australia. | To evaluate the efficacy of using a serious game to teach hazard and safety assessments in community and residential healthcare settings | NS (n=8)  Sex: 100 % female  Age: 18-54 years. | NR | Safe Environments' game. | Qualitative/ Interpretative Phenomenological Analysis of semi-structured interviews | The serious game engages nursing students who reported increased knowledge and stimulation of CT. |
| Weatherspoon & Wyatt [52] (2012)  USA | To determine the feasibility of using a computer-based simulation with senior NS to improve CT | NS (n=23)  Sex: 90-95% female  Age: Median 22 years  CG^8^ (n=12)  IG^7^ (n=11) | Kolb´s Experiential Learning Theory | Computer-based simulation (single-player) | Pre- posttest intervention-experimental design/ Triage Acuity Instrument (TAI) | IG showed a very significant improvement of CJ skills while the CG showed a marginally improvement. The effect size was large, Cohens`d = 0.97 |
| Weatherspoon et al. [55] (2015)  USA | To compare the effects of an electronic interactive simulation (EIS) and traditional paper case study simulation (TPCSS) on the CT disposition and CJ skills of senior baccalaureate NSs | Senior bachelor NS (n=117) divided in EIS (n=60) and TPCSS (n=57)  Sex: 82.9% female  Age: Mean 25.5 years (±6.3) | Kolb´s Experiential Learning Theory | Computer-based Electronic Interactive Simulation (EIS) | RCT with a pre-post-test design/ California Critical Thinking Dispositions Inventory (CCTDI) and the Triage Acuity Instrument (TAI) | Participants who used EIS over a 2-week period increased their scores for CT thinking disposition overall and on the subscales: Truth seeking, Open mindedness and Confidence in Reasoning |
| Williams et al. [92] (2024)  USA | To determine whether virtual patient simulation (VPS) improves clinical reasoning and communication in nursing students | Baccalaureate NS (n=19)  Sex: 94.7% female  Age: Range 18-24 | Experiential Learning Theory (ELT) | Virtual Patient Simulation (VPS) | A quantitative, quasi-experimental design with a pre-test post-test methodology/ The Student Performance Index (SPI) | Statistically significant improvement in clinical reasoning and communication; effective in therapeutic communication and subjective data collection |
| Wood & Toronto [57] (2012) USA | To assess the influence of human patient simulation practice on CT dispositions in a sample of undergraduate NSs | NS (n=85)  Sex: 96% female  Age: Mean 19.4 years | Affective critical thinking dispositions framework | High-Fidelity Human Patient Simulation | Quasi-experimental design/ California Critical Thinking Disposition Inventory (CCTDI) | No between-group differences were found on overall or subscale CCTDI mean scores. Within-group differences for the HPS practice group were significant for overall scores p < 0.05) and the truth-seeking (p < 0.01) and judiciousness or maturity of judgment (p < 0.01) subscales |
| Wotton et al. [107] (2010)  Australia | To evaluate NS’ perceptions of their experiences with three high-fidelity simulations in a clinical nursing course | NS in 3^rd^. year (n=300)  Sex: NR  Age: NR | Cognitive learning theory | High-fidelity simulation with SimMan®. | Quasi-experimental Post-test with qualitative and quantitative approach/ Self-developed questionnaire and Content analyses of qualitative data | Students perceived high-fidelity simulation as beneficial for understanding nursing care and clinical intervention rationales. They highlighted the need to analyze, interpret, and respond to cues as key to maintaining attention, improving task prioritization, comprehension, and decision-making speed |
| Yang et al. [113] (2024)  China | To evaluate the effect of an integrated non‐immersive virtual and high‐fidelity face-to-face simulation for CJ training. | NS in 3^rd^. year (n=122)  Sex: 86% female  Age: Range 19-24 years  IG (n=61)  CG (n=61) | NR | vSim for Nursing scenarios (Integrated Virtual simulation) and High-fidelity simulation (Face-to-Face) | Mixed-methods, non-randomized, pre-post design/ Lasater Clinical Judgment Rubric and Content analyses of focus group interviews | Integrated simulation improved CJ more effectively than face-to-face simulation alone; qualitative data confirmed quantitative findings |
| Yeh & Chen [134] (2005)  Taiwan | To examine the effects of an educational program with interactive videodisc systems (IVS) in improving affective dispositions toward CT for RN- Bachelor of Science in Nursing students | NS in 2 ^nd^. year from a medical-surgical course (n =126)  Sex: NR  Age: mean of 24.7 years, range 21-49 | NR | Interactive videodisc systems (IVS) | Pre- and post-test quasi-experimental research design/ California Critical Thinking Dispositions Inventory (CCTDI) | After the IVS program, significant improvements were observed in all critical thinking dispositions except inquisitiveness, and all means for the seven dispositions toward CT reach the standard levels except for truth-seeking. Truth-seeking dispositions are significantly improved |

^1^NS: Nursing Student; ^2^IG: Intervention Group; ^3^NR: Not Reported; ^4^CT: Critical Thinking; ^5^SG: Simulation Group: ^6^WCSG: Written Case Studies Group; ^7^CR: Clinical Reasoning; ^8^CG: Control Group; ^9^RCT: Randomized controlled trial; ^10^VR: Virtual Reality; ^11^CD-M: Clinical Decision-Making; ^12^CJ: Clinical Judgment.

**Reference details:**

51. Blakeslee JR. Effects of high-fidelity simulation on the critical thinking skills of baccalaureate nursing students: A causal-comparative research study. Nurse Education Today. 2020;92:104494. PMID: 32544764. doi: 10.1016/j.nedt.2020.104494.

52. Weatherspoon DL, Wyatt TH. Testing computer-based simulation to enhance clinical judgment skills in senior nursing students. Nursing Clinics. 2012;47(4):481-91. PMID: 23137600. doi: 10.1016/j.cnur.2012.07.002.

53. Donovan LM, Argenbright CA, Mullen LK, Humbert JL. Computer-based simulation: Effective tool or hindrance for undergraduate nursing students? Nurse Educ Today. 2018 Oct;69:122-7. PMID: 30048812. doi: 10.1016/j.nedt.2018.07.007.

54. Fero LJ, O’Donnell JM, Zullo TG, Dabbs AD, Kitutu J, Samosky JT, et al. Critical thinking skills in nursing students: Comparison of simulation‐based performance with metrics. Journal of advanced nursing. 2010;66(10):2182-93. PMID: 20636471 doi: 10.1111/j.1365-2648.2010.05385.x.

55. Weatherspoon DL, Phillips K, Wyatt TH. Effect of electronic interactive simulation on senior bachelor of science in nursing students' critical thinking and clinical judgment skills. Clinical Simulation in Nursing. 2015;11(2):126-33. doi: 10.1016/j.ecns.2014.11.006.

56. Brown D, Chronister C. The effect of simulation learning on critical thinking and self-confidence when incorporated into an electrocardiogram nursing course. Clinical Simulation in Nursing. 2009;5(1):e45-e52. doi: 10.1016/j.ecns.2008.11.001.

57. Wood RY, Toronto CE. Measuring critical thinking dispositions of novice nursing students using human patient simulators. Journal of Nursing Education. 2012;51(6):349-52. PMID: 22533501. doi: 10.3928/01484834-20120427-05.

58. Rush KL, Dyches CE, Waldrop S, Davis A. Critical thinking among RN-to-BSN distance students participating in human patient simulation. Journal of Nursing Education. 2008;47(11):501-7. PMID: 19010048. doi: 10.3928/01484834-20081101-07.

59. Lowdermilk DL, Fishel AH. Computer simulations as a measure of nursing students' decision-making skills. 1991;30(1):34-9. PMID: 1847408. doi: 10.3928/0148-4834-19910101-09.

60. Ravert P. Patient simulator sessions and critical thinking. Journal of Nursing Education. 2008;47(12):557-62. PMID: 19112746. doi: 10.3928/01484834-20081201-06.

61. Everett-Thomas R, Joseph L, Trujillo G. Using virtual simulation and electronic health records to assess student nurses' documentation and critical thinking skills. Nurse education today. 2021;99:104770. PMID: 33516978. doi: 10.1016/j.nedt.2021.104770.

62. Schoening AM, Sittner BJ, Todd MJ. Simulated clinical experience: Nursing students' perceptions and the educators' role. Nurse educator. 2006;31(6):253-8. PMID: 17108788 doi: 10.1097/00006223-200611000-00008.

63. Mahoney AED, Hancock LE, Iorianni-Cimbak A, Curley MA. Using high-fidelity simulation to bridge clinical and classroom learning in undergraduate pediatric nursing. Nurse education today. 2013;33(6):648-54. doi: 10.1016/j.nedt.2012.01.005.

64. Goodstone L, Goodstone MS, Cino K, Glaser CA, Kupferman K, Dember-Neal T. Effect of simulation on the development of critical thinking in associate degree nursing students. Nursing education perspectives. 2013;34(3):159-62. PMID: 23914457. doi: 10.5480/1536-5026-34.3.159.

65. Sullivan-Mann J, Perron CA, Fellner AN. The effects of simulation on nursing students' critical thinking scores: A quantitative study. Newborn and Infant Nursing Reviews. 2009;9(2):111-6. doi: 10.1053/j.nainr.2009.03.006.

66. Parr MB, Sweeney NM. Use of human patient simulation in an undergraduate critical care course. Critical Care Nursing Quarterly. 2006;29(3):188-98. PMID: 16862020. doi: 10.1097/00002727-200607000-00003.

67. Fogg N, Kubin L, Wilson CE, Trinka M. Using virtual simulation to develop clinical judgment in undergraduate nursing students. Clinical Simulation in Nursing. 2020;48:55-8. doi: 10.1016/j.ecns.2020.08.010.

68. Powell-Laney S, Keen C, Hall K. The Use of Human Patient Simulators to Enhance Clinical Decision-making of Nursing Students. Education for Health. 2012;25(1):11-5. PMID: 23787379. doi: 10.4103/1357-6283.99201.

69. Smith A, Lollar J, Mendenhall J, Brown H, Johnson P, Roberts S. Use of multiple pedagogies to promote confidence in triage decision making: a pilot study. Journal of Emergency Nursing. 2013;39(6):660-6. PMID: 22421315. doi: 10.1016/j.jen.2011.12.007.

70. Burns HK, O'Donnell J, Artman J. High-fidelity simulation in teaching problem solving to 1st-year nursing students: A novel use of the nursing process. Clinical Simulation in Nursing. 2010;6(3):e87-e95. doi: 10.1016/j.ecns.2009.07.005.

71. Lewis DY, Ciak AD. THE IMPACTof aSimulation Lab ExperienceforNursing Students. Nursing education perspectives. 2011;32(4):256-8. PMID: 21923007.

72. Kiernan LC, Olsen DM. Improving clinical competency using simulation technology. Nursing. 2020;50(7):14. PMID: 32472824 doi: 10.1097/01.nurse.0000668448.43535.4f.

73. Rhodes ML, Curran C. Use of the human patient simulator to teach clinical judgment skills in a baccalaureate nursing program. CIN: Computers, Informatics, Nursing. 2005;23(5):256-62. PMID: 16166827. doi: 10.1097/00024665-200509000-00009.

74. Hudson S, Penkalski MR. High-Fidelity Simulation Versus Case Study: Which Is Best for Practical Nursing Students? Nursing Education Perspectives. 2022;43(1):49-50. PMID: 34939771. doi: 10.1097/01.nep.0000000000000767.

75. Pardue KT, Holt K, Dunbar D-M, Baugh N. Exploring the Development of Nursing Clinical Judgment Among Students Using Virtual Reality Simulation. Nurse Educator. 2023;48(2):71-5. PMID: 36332204. doi: 10.1097/nne.0000000000001318.

76. Badowski D, Rossler KL, Reiland N. Exploring student perceptions of virtual simulation versus traditional clinical and manikin-based simulation. Journal of Professional Nursing. 2021;37(4):683-9. PMID: 34187664. doi: 10.1016%2Fj.profnurs.2021.05.005.

77. Cieslowski B, Haas T, Oh KM, Chang K, Oetjen CA. The Development and Pilot Testing of Immersive Virtual Reality Simulation Training for Prelicensure Nursing Students: A Quasi-Experimental Study. Clinical Simulation in Nursing. 2023;77:6-12. doi: 10.1016/j.ecns.2023.02.001.

78. Thiele JE, Baldwin JH, Hyde RS, Sloan B, Strandquist GA. An investigation of decision theory: what are the effects of teaching cue recognition? 1986;25(8):319-24. PMID: 3023568. doi: 10.3928/0148-4834-19861001-05.

79. Powers K, Neustrup W, Thomas C, Saine A, Sossoman LB, Ferrante-Fusilli FA, et al. Baccalaureate nursing students' experiences with multi-patient, standardized patient simulations using telehealth to collaborate. Journal of Professional Nursing. 2020;36(5):292-300. PMID: 33039061. doi: 10.1016/j.profnurs.2020.03.013.

80. Montenery SM, Walker M, Sorensen E, Thompson R, Kirklin D, White R, et al. Millennial generation student nurses’ perceptions of the impact of multiple technologies on learning. Nursing education perspectives. 2013;34(6):405-9. PMID: 24475603. doi: 10.5480/10-451.

81. Lasater K. High-fidelity simulation and the development of clinical judgment: Students' experiences. Journal of Nursing education. 2007;46(6):269-76. PMID: 17580739. doi: 10.3928/01484834-20070601-06.

82. Henrichs B, Rule A, Grady M, Ellis W. Nurse anesthesia students' perceptions of the anesthesia patient simulator: a qualitative study. AANA journal. 2002;70(3):219-25. PMID: 12078470.

83. Kleinheksel A. Transformative learning through virtual patient simulations: predicting critical student reflections. Clinical Simulation in Nursing. 2014;10(6):e301-e8. doi: 10.1016/j.ecns.2014.02.001.

84. Kuiper R, Heinrich C, Matthias A, Graham MJ, Bell-Kotwall L. Debriefing with the OPT model of clinical reasoning during high fidelity patient simulation. International Journal of Nursing Education Scholarship. 2008;5(1):000010220215489231466. PMID: 18454731. doi: 10.2202/1548-923x.1466.

85. Aller L, Shelestak D, Phillips L, Reed J, Allen B. Measuring nursing student development through computer-based simulation activities. Nurse Educator. 2023;48(6):298-303. doi: 10.1097/NNE.0000000000001423.

86. Beebe SL, McNelis AM, El-Banna M, Dreifuerst KT, Zhou QP. Nailing the Diagnosis: Using Screen-Based Simulation to Improve Factors of Diagnostic Reasoning in Family Nurse Practitioner Education. Clinical Simulation in Nursing. 2024;91:101528. doi: 10.1016/j.ecns.2024.101528.

87. Cole B. Enhancing clinical reasoning and student confidence through pediatric simulation. Journal of Pediatric Nursing. 2024;78:e432-e7. doi: 10.1016/j.pedn.2024.08.005.

88. Elliott EJ, Firkins JL. Use of Scenario-based Activities for Learning and Confidence in Accelerated Bachelor Nursing Students in Acute Settings. International Journal of Nursing Education. 2024;16(4). doi: 10.37506/ps1p9p93.

89. Fogg N, Yousef MG, Thompson A, Bauman EB, Kardong-Edgren S. Fostering clinical decision-making using screen-based simulations aligned with the NCSBN Clinical Judgment Measurement Model. Clinical Simulation in Nursing. 2023;84:101452. doi: 10.1016/j.ecns.2023.101452.

90. Legge TR, Holthaus A, Hallmark B, Alexander K. Preparing for the next generation NCLEX using a virtual simulation. Journal of Nursing Education. 2024;63(7):485-9. doi: 10.3928/01484834-20230713-01.

91. Perez A, Andrews A, Luebbert R. Differences in community health nursing learning outcomes based on simulation modality. Clinical Simulation in Nursing. 2024;96:101606. doi: 10.1016/j.ecns.2024.101606.

92. Williams R, Helmer B, Elliott A, Robinson D, Jimenez FA, Faragher ME. Navigating the Virtual Frontier: A Virtual Patient Simulation Pilot Study in Prelicensure Baccalaureate Nursing Education. Clinical Simulation in Nursing. 2024;94:101589. doi: 10.1016/j.ecns.2024.101589.

93. Kang SJ, Hong CM, Lee H. The impact of virtual simulation on critical thinking and self-directed learning ability of nursing students. Clinical Simulation in Nursing. 2020;49:66-72. doi: 10.1016/j.ecns.2020.05.008.

94. Kim JY, Kim EJ. Effects of Simulation on Nursing Students' Knowledge, Clinical Reasoning, and Self-confidence: A Quasi-experimental Study. Korean Journal of Adult Nursing. 2015;27(5):604-11. doi: 10.7475/kjan.2015.27.5.604.

95. Lee H, Han J-W. Development and evaluation of a virtual reality mechanical ventilation education program for nursing students. BMC Medical Education. 2022;22(1):775. PMID: 36357886. doi: 10.1186%2Fs12909-022-03834-5.

96. Park S, Hur HK, Chung C. Learning effects of virtual versus high-fidelity simulations in nursing students: a crossover comparison. BMC nursing. 2022;21(1):100. PMID: 35473614. doi: 10.1186/s12912-022-00878-2.

97. Rim D, Shin H. Development and assessment of a multi-user virtual environment nursing simulation program: A mixed methods research study. Clinical Simulation in Nursing. 2022;62:31-41. doi: 10.1016/j.ecns.2021.10.004.

98. Jeong Y, Lee H, Han JW. Development and evaluation of virtual reality simulation education based on coronavirus disease 2019 scenario for nursing students: A pilot study. Nursing Open. 2022;9(2):1066-76. PMID: 34851042 doi: 10.1002/nop2.1145.

99. Jung MJ, Roh YS. Mediating effects of cognitive load on the relationship between learning flow and clinical reasoning skills in virtual simulation learning. Clinical Simulation in Nursing. 2022;64:16-23. doi: 10.1016/j.ecns.2021.12.004.

100. Shin H, Ma H, Park J, Ji ES, Kim DH. The effect of simulation courseware on critical thinking in undergraduate nursing students: Multi-site pre-post study. Nurse education today. 2015;35(4):537-42. doi: 10.1016/j.nedt.2014.12.004.

101. Kim GM, Lim JY, Kim EJ, Yeom M. Impact of Virtual Reality Mental Health Nursing Simulation on Nursing Students’ Competence. Journal of Multidisciplinary Healthcare. 2024:191-202. doi: 10.2147/jmdh.s435986.

102. Lee E, Baek G. Development and effects of a virtual reality simulation nursing education program combined with clinical practice based on an information processing model. CIN: Computers, Informatics, Nursing. 2023;41(12):1016-25. doi: 10.1097/cin.0000000000001051.

103. Lee E, Baek G, Hwang Y. Effectiveness of the Patient’s Severity Classification Competency Promotion Virtual Reality Program of Nursing Students during the COVID-19 Pandemic Period. Healthcare. 2023;11(8):1122. doi: 10.3390/healthcare11081122.

104. Lee E, Baek G. Development and Effects of Adult Nursing Education Programs Using Virtual Reality Simulations. Healthcare. 2024;12(13):1313. doi: 10.3390/healthcare12131313.

105. Peddle M, Mckenna L, Bearman M, Nestel D. Development of non-technical skills through virtual patients for undergraduate nursing students: An exploratory study. Nurse education today. 2019;73:94-101. PMID: 30610960. doi: 10.1016/j.nedt.2018.11.008.

106. Frost J, Delaney L, Fitzgerald R. Exploring the application of mixed reality in nurse education. BMJ Simulation & Technology Enhanced Learning. 2020;6(4):214-9. PMID: 35520006 doi: 10.1136/bmjstel-2019-000464.

107. Wotton K, Davis J, Button D, Kelton M. Third-year undergraduate nursing students’ perceptions of high-fidelity simulation. Journal of Nursing Education. 2010;49(11):632-9. PMID: 20795614. doi: 10.3928/01484834-20100831-01.

108. Volejnikova-Wenger S, Andersen P, Clarke K-A. Student nurses' experience using a serious game to learn environmental hazard and safety assessment. Nurse Education Today. 2021;98:104739. PMID: 33418087. doi: 10.1016/j.nedt.2020.104739.

109. Finn J, Bradley L. vSim® gerontology and inquiry-based learning enhancing clinical reasoning and preparation for practice. Teaching and Learning in Nursing. 2023;18(4):e146-e50. doi: 10.1016/j.teln.2023.05.002.

110. Kiegaldie D, Shaw L. Virtual reality simulation for nursing education: effectiveness and feasibility. BMC nursing. 2023;22(1):488. doi: 10.1186/s12912-023-01639-5.

111. Luo Y, Geng C, Pei X, Chen X, Zou Z. The evaluation of the distance learning combining webinars and virtual simulations for senior nursing students during the COVID-19 period. Clinical Simulation in Nursing. 2021;57:31-40. PMID: 35915811. doi: 10.1016/j.ecns.2021.04.022.

112. Han F, Jin S. Evaluation of the combination of virtual simulation and in-person simulation among undergraduate nursing students: A mixed methods study. Nurse Education in Practice. 2024;75:103899. doi: 10.1016/j.nepr.2024.103899.

113. Yang J, Zhou WJ, Zhou SC, Luo D, Liu Q, Wang A-L, et al. Integrated virtual simulation and face-to-face simulation for clinical judgment training among undergraduate nursing students: a mixed-methods study. BMC Medical Education. 2024;24(1):32. doi: 10.1186/s12909-023-04988-6.

114. Li G, Gao L, Yin H, Jia Y, Zhang X, Tian H, et al. Implementation and Evaluation of a Virtual Reality-Based Cognitive Assessment and Rehabilitation Simulation Course in Undergraduate Nursing Students: A Pre-Post Study. Clinical Simulation in Nursing. 2023;81:101430. doi: 10.1016/j.ecns.2023.101430.

115. Li Y, Lv Y, Dorol RD, Wu J, Ma A, Liu Q, et al. Integrative virtual nursing simulation in teaching cardiopulmonary resuscitation: A blended learning approach. Australasian Emergency Care. 2024;27(1):37-41. doi: 10.1016/j.auec.2023.07.006.

116. Tong LK, Li YY, Au ML, Ng WI, Wang SC, Liu Y, et al. The effects of simulation-based education on undergraduate nursing students' competences: a multicenter randomized controlled trial. BMC nursing. 2024;23(1):400. doi: 10.1186/s12912-024-02069-7.

117. Şahin G, Başak T. The Effect of Virtual Patient Simulation on Nursing Students' Clinical Decision Making and Problem-Solving Skills. Journal of Education & Research in Nursing/Hemşirelikte Eğitim ve Araştırma Dergisi. 2021;18(2):178-82. doi: 10.5152/jern.2021.26504.

118. Durmaz A, Dicle A, Cakan E, Cakir S. Effect of screen-based computer simulation on knowledge and skill in nursing students’ learning of preoperative and postoperative care management: a randomized controlled study. CIN: Computers, Informatics, Nursing. 2012;30(4):196-203. doi: 10.1097/NCN.0b013e3182419134.

119. Karaduman GS, Basak T. Is Virtual Patient Simulation Superior to Human Patient Simulation: A Randomized Controlled Study. CIN: Computers, Informatics, Nursing. 2023;41(6):467-76. PMID: 36633879. doi: 10.1097/cin.0000000000000957.

120. Edeer AD, Sarikaya A. Views, perceptions and recommendations of nursing students on screen-based computer simulation: Qualitative. International Journal of Psychology and Educational Studies. 2018;5(2):21-9. doi: 10.17220/ijpes.2018.02.003.

121. Calik A, Kapucu S. Comparative Effectiveness of Developed Serious Game Versus Standardized Patients' Simulation in Nursing Education. Games for health journal. 2024. doi: 10.1089/g4h.2024.0037.

122. Onay T, Gulpinar MA, Saracoglu M, Akdeniz E. Determining the effectiveness of a virtual service/patient-based education program on patient care and clinical decision-making in nursing: A quasi-experimental study. Northern Clinics of Istanbul. 2024;11(5):422-33. doi: 10.14744/nci.2024.78095.

123. Chircop A, Cobbett S, Boudreau C, Egert A, Filice S, Harvey A, et al. Multi-Jurisdictional Evaluation of Sentinel City Virtual Simulation for Community Health Nursing Clinical Education. Quality Advancement in Nursing Education-Avancées en formation infirmière. 2022;8(4):2. doi: 10.17483/2368-6669.1352.

124. Hamidi Y, Tyerman JJ, Domingue J-L, Luctkar-Flude M. The perceived effectiveness of a suicide assessment virtual simulation module for undergraduate nursing students. Clinical Simulation in Nursing. 2024;89:101509. doi: 10.1016/j.ecns.2024.101509.

125. Maheu-Cadotte M-A, Dubé V, Lavoie P. Development and Contribution of a Serious Game to Improve Nursing Students' Clinical Reasoning in Acute Heart Failure: A Multimethod Study. CIN: Computers, Informatics, Nursing. 2023;41(6):410-20. doi: 10.1097/CIN.0000000000000966.

126. Vihos J, Chute A, Carlson S, Shah M, Buro K, Velupillai N. Virtual Reality Simulation in a Health Assessment Laboratory Course: A Mixed-methods Explanatory Study Examining Student Satisfaction and Self-confidence. Nurse Educator. 2024:10.1097. doi: 10.1097/NNE.0000000000001635.

127. Koivisto J-M, Multisilta J, Niemi H, Katajisto J, Eriksson E. Learning by playing: A cross-sectional descriptive study of nursing students' experiences of learning clinical reasoning. Nurse education today. 2016;45:22-8. PMID: 27429399 doi: 10.1016/j.nedt.2016.06.009.

128. Koivisto J-M, Haavisto E, Niemi H, Katajisto J, Multisilta J. Elements explaining learning clinical reasoning by playing simulation game. International Journal of Serious Games. 2016;3(4):29-43. doi: 10.17083/ijsg.v3i4.136.

129. Havola S, Haavisto E, Mäkinen H, Engblom J, Koivisto J-M. The effects of computer-based simulation game and virtual reality simulation in nursing students' self-evaluated clinical reasoning skills. CIN: Computers, Informatics, Nursing. 2021;39(11):725-35. PMID: 33941719. doi: 10.1097/cin.0000000000000748.

130. Elcokany NM, Abdelhafez AI, Samuel Sharaby VM, Belal S. Use of computer-based scenarios for clinical teaching: Impact on nursing students’ decision-making skills. Healthcare. 2021;9(9):1228. PMID: 34575002. doi: 10.3390/healthcare9091228.

131. Albagawi B, Alsalamah Y, Alharbi M, Alrawili R, Babkair LA, Allari R, et al. The Lived Experiences of Saudi Nursing Students in Digital Clinical Experience: A Phenomenological Study. Cureus. 2024;16(2). doi: 10.7759/cureus.53830.

132. Alharbi HF, Alsubaie A, Gharawi R, Mazroo RB, Alajaleen S, Alsultan M, et al. The relationship between virtual simulation, critical thinking, and self-directed learning abilities of nursing students in Riyadh, Saudi Arabia. PeerJ. 2024;12:e18150. doi: 10.7717/peerj.18150.

133. Hwang GJ, Chang CY. Facilitating decision-making performances in nursing treatments: a contextual digital game-based flipped learning approach. Interactive Learning Environments. 2020;31(1):156-71. doi: 10.1080/10494820.2020.1765391.

134. Yeh M-L, Chen H-H. Effects of an educational program with interactive videodisc systems in improving critical thinking dispositions for RN-BSN students in Taiwan. International Journal of Nursing Studies. 2005;42(3):333-40. PMID: 15708020. doi: 10.1016/j.ijnurstu.2004.06.008.

135. PENG Y-C, WU H-S. The Efficacy of High-Fidelity Telesimulation in Preparing Nursing Students for Emergency and Critical Patient Care: A Prospective Study. Journal of Nursing Research. 2024;32(5):e348. doi: 10.1097/jnr.0000000000000631.

136. Blanié A, Amorim M-A, Benhamou D. Comparative value of a simulation by gaming and a traditional teaching method to improve clinical reasoning skills necessary to detect patient deterioration: a randomized study in nursing students. BMC medical education. 2020;20(1):1-11. PMID: 32075641. doi: 10.1186/s12909-020-1939-6.

137. Fung JTC, Zhang W, Yeung MN, Pang MTH, Lam VSF, Chan BKY, et al. Evaluation of students' perceived clinical competence and learning needs following an online virtual simulation education programme with debriefing during the COVID‐19 pandemic. Nursing Open. 2021;8(6):3045-54. PMID: 34331397. doi: 10.1002/nop2.1017.

138. Manik M, Gultom E, Sibuea R, Pailak H. Virtual Simulation Learning from Indonesian Nursing Students’ Perspectives. Open Access Macedonian Journal of Medical Sciences (OAMJMS). 2022;10(G):112-7. doi: 10.3889/oamjms.2022.8239.

139. Hosseini TM, Ahmady S, Edelbring S. Teaching Clinical Decision-Making Skills to Undergraduate Nursing Students via Web-based Virtual Patients during the COVID-19 Pandemic: A New Approach to The CyberPatient TM Simulator. Journal of Contemporary Medical Sciences. 2022;8(1):31-7. doi: 10.22317/jcms.v8i2.1158.

140. Dubovi I. Online computer-based clinical simulations: the role of visualizations. Clinical Simulation in Nursing. 2019;33:35-41. doi: 10.1016/j.ecns.2019.04.009.

141. Fawaz MA, Hamdan-Mansour AM. Impact of high-fidelity simulation on the development of clinical judgment and motivation among Lebanese nursing students. Nurse education today. 2016;46:36-42. doi: 10.1016/j.nedt.2016.08.026.

142. Reierson IÅ, Solli H, Bjørk IT. Nursing students' perspectives on telenursing in patient care after simulation. Clinical Simulation in Nursing. 2015;11(4):244-50. doi: 10.1016/j.ecns.2015.02.003.

143. Padilha JM, Machado PP, Ribeiro A, Ramos J, Costa P. Clinical virtual simulation in nursing education: randomized controlled trial. Journal of medical Internet research. 2019;21(3):e11529. PMID: 30882355. doi: 10.2196/11529.

144. Sterner A, Sköld R, Andersson H. Effects of blended simulation on nursing students’ critical thinking skills: A quantitative study. SAGE open nursing. 2023;9:23779608231177566. doi: 10.1177/23779608231177566.

145. Uppor W, Klunklin A, Viseskul N, Skulphan S. Effects of Experiential Learning Simulation-Based Learning Program on Clinical Judgment Among Obstetric Nursing Students. Clinical Simulation in Nursing. 2024;92:101553. doi: 10.1016/j.ecns.2024.101553.

146. McCaughey CS, Traynor MK. The role of simulation in nurse education. Nurse education today. 2010;30(8):827-32. doi: 10.1016/j.nedt.2010.03.005.
